# Quantifying the effects of cultivation mode and sprouting stage on tea bud morphology and chemical quality

**DOI:** 10.3389/fpls.2026.1747538

**Published:** 2026-01-30

**Authors:** Wei Zhu, Jiayi Yang, Suqing Xiao, Wenjing Hu, Jiamin Tian, Yuchen Zeng, Zhiming Huang, Ziyi Yuan

**Affiliations:** 1 Jingdezhen Key Laboratory of Tea Plant Germplasm Resources and Ecological Cultivation, Jingdezhen University, Jingdezhen, China; 2 Ministry of Education Key Laboratory of Silviculture and Conservation, Beijing Forestry University, Beijing, China; 3 Fuliang Changnan Tea Co., Ltd., Jingdezhen, China

**Keywords:** cultivation modes, sprouting dynamics, tea bud morphology, chemical quality, correlation

## Abstract

While the influences of environment and genetics on plant traits are widely recognized, quantitative analysis of their relative contributions remains limited in crop systems, constraining precise environmental manipulation of agronomic traits. This study investigated the effects of cultivation mode (representing external environment) and sprouting stage (reflecting internal developmental program) on tea bud morphology and chemical quality, with particular focus on their intrinsic relationships. Through dynamic monitoring at three sprouting period across five contrasting cultivation modes in Fuliang County, Jiangxi Province, China, key morphological and chemical traits were obtained. The effects of cultivation mode and sprouting stage were assessed using linear mixed-effects models and partial ω². Results revealed a consistent developmental trend over the observed sprouting period: across all cultivation modes, bud length and number increased continuously, while width decreased, and size first declined then increased. Critically, cultivation mode had a stronger influence on bud width, number, and size (effect sizes: 0.739—0.768) than on length (0.132), which was predominantly governed by the sprouting stage (effect size: 0.812). Cultivation mode exerted a stronger effect on morphological composite traits (0.753) than on chemical composite traits (0.530). The correlations between bud length and width and between width and number shifted from positive to negative during sprouting (*P* < 0.05), suggesting increasing resource competition among these traits. Most importantly, no significant correlations were observed between morphological traits and chemical quality at the final sprouting stage. Based on data from this single yet harvest-critical time point, our results directly challenge the traditional belief that bud size predicts tea quality. These findings provide new insights into gene-environment interactions, phenotypic plasticity, and ecological trade-offs in agroecosystems, establishing a scientific basis for precision management strategies that simultaneously optimize tea production and quality.

## 1 Introduction

Plant traits represent the ultimate phenotype resulting from the interaction between genotype and environmental factors. The mechanisms underlying their formation constitute a central topic in genetics, ecology, and crop breeding (Chapin et al., 1987; Lynch and Walsh, 1998). Disentangling the relative contributions of intrinsic developmental programs and external environments to trait formation is not only crucial for understanding species adaptation and ecological differentiation (Tsukaya, 2005; Carbone et al., 2009; Li et al., 2020b), but also provides a theoretical foundation for the precise regulation of agricultural traits. However, in many crop systems, knowledge regarding the quantitative decomposition of their effects and the inherent trade-off relationships remains limited (Wachira et al., 2002; Dutta et al., 2010; Owuor et al., 2011; Hua et al., 2022). This significantly constrains our ability to optimize agronomic traits through targeted environmental management.

Tea (*Camellia sinensis*), as one of the world’s most important economic crops, is cultivated in over 60 countries and regions and consumed by more than 2 billion people globally, demonstrating a profound global influence (International Institute for Sustainable Development, 2024). In premium tea production, harvesting standards such as “a single bud”, “one bud with one leaf”, and “one bud with two leaves” are widely adopted (Aaqil et al., 2023). Tea bud size is a critical agronomic and biological trait, not only directly affecting fresh leaf yield but also closely related to the processing suitability and quality of the final tea product (Li et al., 2020a; An et al., 2021). Therefore, the morphogenesis of tea buds and the formation of their chemical quality are core processes determining economic value. However, existing research has predominantly focused on the macroscopic growth rhythm of tea shoots (Smith et al., 1990; Botwright, 1997; Zhang et al., 2024a) or genetic analyses of bud static traits at specific developmental stages (Zhang et al., 2025b), while systematic investigation into the dynamic developmental patterns of tea bud morphology, their response to cultivation environments, and the correlation mechanisms between morphology and intrinsic chemical quality has long remained insufficient.

In recent years, researchers have begun to employ emerging technologies to explore the genetic basis of tea bud morphogenesis. For instance, Zhang et al. (2025b) utilized digital phenotyping technology to conduct association analysis on a large natural population, revealing extensive variation in tea bud size and providing preliminary insights into its genetic architecture. Such studies offer valuable perspectives for understanding the genetic potential of trait variation. However, this paradigm possesses inherent limitations: relying on static data from a single time point, it fundamentally fails to capture the dynamic trajectory and plastic responses of tea buds during continuous development. More critically, in agricultural ecosystems, phenotypes result from the continuous interaction between genetic programs and environmental signals; systematically deconstructing the effects of cultivation mode (as a manageable environmental factor) and sprouting stage (representing the internal developmental program) on trait formation, and quantitatively comparing their relative importance, is essential for elucidating genotype-by-environment (G×E) interaction mechanisms (Malosetti et al., 2013; El-Soda et al., 2014). Nevertheless, in tea research, the quantitative dissection of the influence of these two factors remains an underexplored area, significantly impeding a deeper understanding of trait formation mechanisms and constraining the optimization of precision cultivation strategies.

The chemical quality of tea is determined by a diverse array of endogenous chemical components and is known to be intricately regulated by multiple factors, including cultivar, season, leaf position, climate, and agronomic practices (Wu et al., 2025). Numerous studies have sought to elucidate the dynamic patterns of these chemical constituents. For example, Li et al. (2016) observed that as leaves mature, carbon and nitrogen metabolic pathways undergo stage-specific shifts, leading to a significant decline in the content of nitrogen-containing compounds such as amino acids. Xu et al. (2021) further demonstrated that gallic acid, caffeine, and certain esterified catechins also decrease with increasing bud and leaf maturity, reflecting a clear developmental stage specificity. At the genetic level, Zhang et al. (2025a) found that the free amino acid content is not only influenced by season and germplasm but also exhibits genetic correlations with sprouting traits, suggesting that quality and morphological traits may share a partial genetic regulatory basis. Although these studies have enhanced our understanding of quality formation from various perspectives, none have directly and systematically examined the intrinsic relationship between the external morphology and internal chemical quality of tea buds. Consequently, the long-held traditional belief in both tea science and industry that “larger buds signify superior quality” still lacks rigorous scientific validation. The persistence of this critical knowledge gap significantly hinders the development of technologies for efficient quality prediction and precision breeding based on appearance traits.

To address the knowledge gaps identified above, this study established five tea garden cultivation modes with significant environmental gradients in Fuliang County, Jiangxi Province, China. Based on the biological principles of tea plant growth and development and the theoretical framework of secondary metabolic regulation, we conducted continuous dynamic monitoring and multi-dimensional quantitative analysis. This research aims to achieve the following three core objectives: (1) investigate the growth dynamics of tea buds under different cultivation modes, quantifying the relative effect size of cultivation mode and sprouting stage on bud morphology. (2) analyze the variation characteristics of chemical quality indicators in tea buds across different cultivation modes, quantify the effect size of cultivation methods on chemical quality, and determine whether cultivation mode has a greater influence on morphological traits or chemical quality. (3) uncover the dynamic correlations among morphological indicators of tea buds, as well as the intrinsic relationships between final bud morphology, growth rate, and chemical quality. By deconstructing the effects of cultivation mode and sprouting stage on tea bud phenotypes, this study will provide quantitative evidence for elucidating genotype-by-environment interaction mechanisms in tea plant trait formation and challenge the conventional belief that “larger buds indicate better quality,” thereby establishing a solid scientific foundation for developing precision agronomic strategies that simultaneously optimize tea yield and quality.

## 2 Materials and methods

### 2.1 Study site and experimental tea plantation

The study site was located in a tea plantation in Fuliang County, Jingdezhen City, Jiangxi Province (E 116°57′--117°42′, N 28°44′--29°56′). The study area experiences a subtropical monsoon climate with distinct seasons and concurrent rain and heat. Precipitation is concentrated in spring and summer, while autumn and winter are mild. The annual average temperature is 17.36°C, with historical extreme maximum temperatures exceeding 40°C and extreme minimum temperatures below -5°C. The average annual precipitation is 1763.5 mm, unevenly distributed in time and space, with the rainy season from April to June accounting for about 46% of the annual total. The terrain is predominantly low mountains and hills, with layered tea terraces, significant diurnal temperature variation, and high air humidity. The soil is primarily red and yellow earth, rich in organic matter and minerals. With a forest coverage rate exceeding 80% and minimal industrial pollution, it is a typical high-quality tea production area, providing an ideal environment for premium tea growth. The experiment was conducted from March to April 2025, encompassing five different cultivation modes, whose basic profiles are shown in **Table 1**.

**Table 1:** List of tea plantations under different cultivation modes.

Cultivation mode	Abbreviation	Altitude (m)	Growth Environment	Tea Cultivar	Intercropped plants and their planting density	Age (years)	Total N (g·kg^−1^)	pH	Air temperature (°C)	Air humidity (%)	Total radiation (W·m^−2^)	Soil moisture (%)
Hilly Tea Monoculture	HT	92.11	Hilltop	*Camellia sinensis* (L.) O. Kuntze cv. Zhuyeqi	—	6	1.65 ± 0.08	4.74 ± 0.04	17.42 ± 3.72	74.58 ± 8.20	150.68 ± 57.61	31.46 ± 2.29
Hilly Tea-Forest Intercropping	HTF	87.77	Hilltop	*C. sinensis* (L.) O. Kuntze cv. Zhuyeqi	Chinese fir, planted in shelterbelts	6	1.75 ± 0.12	4.69 ± 0.21	16.69 ± 3.71	77.96 ± 7.66	107.27 ± 42.23	42.10 ± 1.67
Plain Tea Monoculture	PT	64.53	Riverbank	*C. sinensis* (L.) O. Kuntze cv. Zhuyeqi	—	6	1.79 ± 0.06	5.03 ± 0.05	17.18 ± 3.70	78.32 ± 8.45	188.56 ± 75.19	34.33 ± 3.02
Plain Tea-Forest Intercropping	PTF	65.63	Riverbank	*C. sinensis* (L.) O. Kuntze cv. Zhuyeqi	Cherry trees (canopy width approx. 1.8 m), spaced at 5 m × 20 m	6	1.70 ± 0.03	4.89 ± 0.05	—			
Plain Tea-Soybean Intercropping	PTS	70.03	Riverbank	*C. sinensis* (L.) O. Kuntze cv. Zhuyeqi	Soybean, interplanted within tea rows	6	1.83 ± 0.18	5.63 ± 0.16	—			

The meteorological data in the table represent the mean ± standard deviation during the experimental period from March to April 2025.

### 2.2 Microclimate monitoring in tea plantations

Microclimate data were collected using miniature environmental monitoring systems (ME40, Beijing Shiyu Tong Technology Co., Ltd.). One monitoring device was installed in each of the HT, HTF, and PT tea gardens to continuously track parameters such as air temperature, air humidity, total solar radiation, and topsoil moisture content at the tea canopy level. Data were recorded at 10-minute intervals. After quality control processing, daily average values were calculated. The mean values and standard deviations of each meteorological factor during the entire experimental period (March to April 2025) were compiled for subsequent analysis.

### 2.3 Measurement of tea bud growth dynamics

#### 2.3.1 Monitoring design

*Camellia sinensis* (L.) O. Kuntze cv. Zhuyeqi is an intermediate-sprouting cultivar. In its region of origin, the “one bud with three leaves” stage typically occurs in early April (This cultivar was developed through individual plant selection from the Anhua population by the Tea Research Institute of Hunan Province; see http://hntri.hunaas.cn/?p=30&a=view&r=373 for details). The period from bud dormancy release to the formation of “one bud with three leaves” spans approximately one month, representing the crucial sprouting phase for tea buds. To capture the dynamics during this critical period, we conducted three rounds of bud morphological monitoring, with the final round in early April coinciding with chemical quality assessment. Notably, early April (around the Qingming Festival) also marks the important agricultural milestone when local tea farmers begin harvesting the first batch of premium teas, which primarily consist of single buds. Specifically, three dynamic observations were carried out during the crucial tea bud sprouting period in 2025 on March 6 (the early stage), March 19 (the middle stage), and April 7 (the late stage, proximate to Qingming), across all experimental plots under the five cultivation modes (HT, HTF, PT, PTF, and PTS).

#### 2.3.2 Quadrat setting

for each cultivation mode, five independent observation points (i.e., five biological replicates, n = 5 per mode) were randomly selected. A fixed standard quadrat of 30 cm × 30 cm was established at each point, defining the experimental unit for all subsequent measurements and analyses.

#### 2.3.3 Bud morphology and number

During each observation, the longitudinal (length, mm) and transverse (width, mm) diameters of all vigorous tea buds (with unexpanded apical leaves) within the quadrat were measured using a digital caliper (accuracy: 0.01 mm). Bud size (mm^3^) was expressed as the bud size index, calculated as the square of length multiplied by width. For each quadrat, mean values for length, width, and size were calculated from all measured buds. The total bud count was recorded to obtain the bud number per unit area (buds m^−2^). Growth dynamics curves were constructed based on these three time-series observations.

### 2.4 Determination of tea chemical quality

#### 2.4.1 Sampling and pre-treatment

Sampling was conducted concurrently with the final tea bud growth measurement (April 7). Within the above mentioned fixed quadrats (in section 2.3), all disease-free and fresh tea bud samples were harvested (i.e., five biological replicates per cultivation mode, with at least 80 buds collected per replicate). The samples were immediately placed in an icebox and transported to the laboratory. They were fixed using a microwave oven (medium-high power for 2–2.5 minutes), dried in an oven at 80°C, ground, and sieved through a 40-mesh sieve. The processed powder was sealed and stored protected from light in a -40°C ultra-low temperature freezer for subsequent analysis.

#### 2.4.2 Determination of free amino acids and tea polyphenols

The contents of free amino acids and tea polyphenols were determined according to the Chinese National Standards GB/T 8314–2013 and GB/T 8313-2018, respectively, using a UV spectrophotometer (UV-8000, Shanghai Metash Instruments CO., LTD., Shanghai, China). Samples were extracted with hot water (for amino acid determination) or 70% methanol (for tea polyphenol determination), followed by filtration or centrifugation and volume adjustment. An aliquot of the extract was then reacted with ninhydrin solution or Folin-Ciocalteu reagent, and the absorbance was measured at wavelengths of 570 nm or 765 nm, respectively. Quantification was performed using standard curves prepared with theanine or gallic acid as the standard, respectively.

### 2.5 Data analysis

#### 2.5.1 Part I: effect analysis of cultivation mode and sprouting stage on tea bud morphology and growth rate

To quantify the effects of cultivation mode and sprouting stage on tea bud morphology, we employed linear mixed-effects models for analysis. For the four morphological indicators of tea buds (length, width, number, and size), the model fixed effects included cultivation mode (5 levels: HT, HTF, PT, PTF, PTS), sprouting stage (3 levels: early, middle, late, set as an ordered factor), and their interaction. The random effect was quadrat, to control for the non-independence among repeated measurements from the same quadrat (n = 5). To handle temporal autocorrelation common in repeated measures data, we systematically compared six residual covariance structures: Independent (INDEP), Compound Symmetry (CS), First-Order Autoregressive (AR1), Heterogeneous Compound Symmetry (CSH), Toeplitz (TOEP), and Unstructured (UN). Specific formula expressions can be found in the supplementary material section “1. Model Formulations”.

The optimal covariance structure for each morphological indicator was selected based on the Akaike Information Criterion (AIC) (**Supplementary Table S1**). Model assumption tests (**Supplementary Table S2**) showed that, except for tea bud width residuals not fully meeting the normality assumption (Shapiro-Wilk *p* = 0.023), the residuals for the other indicators satisfied the assumptions of normality and homogeneity of variance. Given that linear mixed models are robust to slight deviations from normality, subsequent inferences were all based on the selected best models. We reported the Type III ANOVA results of the best models (**Supplementary Table S3**), fixed effect coefficient estimates, standard errors and significance (**Supplementary Table S4**), and residual diagnostic plots (**Supplementary Figures S1**–**S4**). Based on the model results, we used the emmeans package for *post-hoc* multiple comparisons, with *p*-values adjusted using the Tukey method. To quantify the relative importance of each factor, we calculated the relative contribution (%) of cultivation mode, sprouting stage, and their interaction to the variation of each morphological indicator, based on the *F*-values from the model Type III ANOVA, and calculated partial ω² as a standardized effect size to indicate the proportion of variance attributable to each factor after accounting for other effects in the model.

To analyze the dynamic growth of the morphological indicators, we calculated the growth rate for each indicator between two adjacent sprouting stages ( $GR_{stage}$), i.e., the daily absolute change:


$$GR_{stage}=\frac{\left(Y_{t+1}-Y_{t}\right)}{\Delta t}$$

where Y represents the measured value of the morphological indicator, and Δt is the number of days between adjacent stages (early to middle: 13 days, middle to late: 19 days). Subsequently, we used paired *t*-tests to compare the differences in growth rates for each indicator between the “early–middle” and “middle–late” stages (**Supplementary Table S5**, **Supplementary Figure S5**).

Calculation of overall growth rate: To obtain a single indicator characterizing the growth intensity over the entire sprouting process (early to late) for subsequent correlation analysis in Part II, we calculated the time-weighted average overall growth rate ( OverallGR). This method considered the influence of different stage durations, avoiding bias from simple arithmetic averaging. The calculation formula is as follows:


$$OverallGR=\frac{\left(GR_{early-mid}\times \Delta t_{early-mid}\right)+\left(GR_{mid-late}\times \Delta t_{mid-late}\right)}{\left(\Delta t_{early-mid}+\Delta t_{mid-late}\right)}$$

where $GR_{early-mid}$ and $GR_{mid-late}$ are the growth rates for the early-middle and middle-late stages, respectively, $\Delta t_{early-mid}$ = 13 days, $\Delta t_{mid-late}$ = 19 days, $\Delta t_{total}$ = 32 days. The calculation was based on the raw data from each quadrat, ultimately obtaining the overall growth rates for length, width, number, and size for each quadrat replicate over the entire sprouting period.

#### 2.5.2 Part II: effect analysis of cultivation mode on chemical quality, final morphology, and growth rate

To assess the comprehensive impact of cultivation mode on the chemical quality of mature tea buds, their final morphology, and the overall growth rate during the sprouting period, we established a series of linear mixed models. The model fixed effect was cultivation mode, and the random effect was quadrat (n = 5), used to analyze the following three categories of indicators: 1. Chemical quality indicators: Free amino acid content, tea polyphenol content, and the polyphenol-to-amino acid ratio determined at the late sprouting stage. 2. Final morphological indicators: Tea bud length, width, number, and size measured at the late sprouting stage. 3. Overall growth rate indicators: The overall growth rates for each morphological indicator over the entire sprouting period, calculated based on the method from Part I.

All models underwent diagnostic checks for residual normality (Shapiro-Wilk test) and homogeneity of variance (Levene’s test) (**Supplementary Table S6**), and residual diagnostic plots were provided (**Supplementary Figures S6**-**S16**). Based on models meeting the basic assumptions, we performed Type III ANOVA to test the main effect of cultivation mode (**Supplementary Table S7**) and reported the model parameter estimates table (**Supplementary Table S8**). Simultaneously, we calculated the partial ω² for cultivation mode’s effect on each indicator as a measure of its effect size.

To comprehensively evaluate the overall influence intensity of cultivation mode on each set of indicators, we performed Principal Component Analysis (PCA). PCA was conducted separately on the chemical indicators, morphological indicators, and growth rate indicator, and the scores of the first principal component (PC1) were extracted. These scores were named the “Chemical Composite Index,” “Morphological Composite Index,” and “Growth Composite Index,” respectively, summarizing most of the variation within each category (variance explained > 60%, **Supplementary Tables S9**, **S10**). Subsequently, we again used linear mixed models (fixed effect: cultivation mode, random effect: quadrat) to analyze the effect of cultivation mode on these composite indices (**Supplementary Tables S7**, **S8**, **Supplementary Figures S17**, **S19**) and calculated the partial ω², thereby comparing the differences in the influence of cultivation mode at a comprehensive, dimension-reduced level.

#### 2.5.3 Part III: inter-indicator correlations

To explore the intrinsic relationships among various indicators and their dynamic changes during the sprouting process, we conducted systematic non-parametric correlation analyses. First, we analyzed the pairwise correlations among the three morphological indicators (length, width, number) within each sprouting stage. Second, we performed a comprehensive correlation analysis on all indicators involved in Part II (3 late-stage chemical quality indicators, 4 late-stage morphological indicators, 4 overall growth rate indicators).

As some indicator data distributions did not conform to normality based on the Shapiro-Wilk test (**Supplementary Tables S11**, **S12**), and scatter plots suggested potential non-linear trends for some variable pairs, we chose Spearman’s rank correlation coefficient ( $\rho_{s}$) for assessment. This method does not rely on data distribution assumptions and is robust to outliers.

To test whether the correlation coefficient for the same pair of morphological indicators (e.g., length *vs*. width) differed statistically between different sprouting stages (e.g., early *vs*. middle), we employed a non-parametric Bootstrap permutation test. The specific steps were: 1) Combine the data from the two stages; 2) Perform random resampling with replacement to create Bootstrap samples (1000 repetitions); 3) For each repetition, calculate the difference in $\rho_{s}$ between the two stages based on the Bootstrap samples; 4) Calculate the 95% confidence interval based on the distribution of the differences. If this interval did not contain 0, the correlation coefficients for the two stages were considered significantly different (*P* < 0.05; **Supplementary Table S13**). This method avoids the strict data distribution requirements of parametric tests.

All statistical analyses and graphics were generated using R software (v. 4.5.2, R Core Team, 2025). The following packages were used: nlme, lme4, lmerTest, emmeans, tidyverse, ggplot2, MuMIn, car, performance, effectsize, FactoMineR, factoextra, psych, and cocor.

## 3 Results

### 3.1 Tea bud growth followed similar dynamic trends across cultivation modes

**Figure 1:**
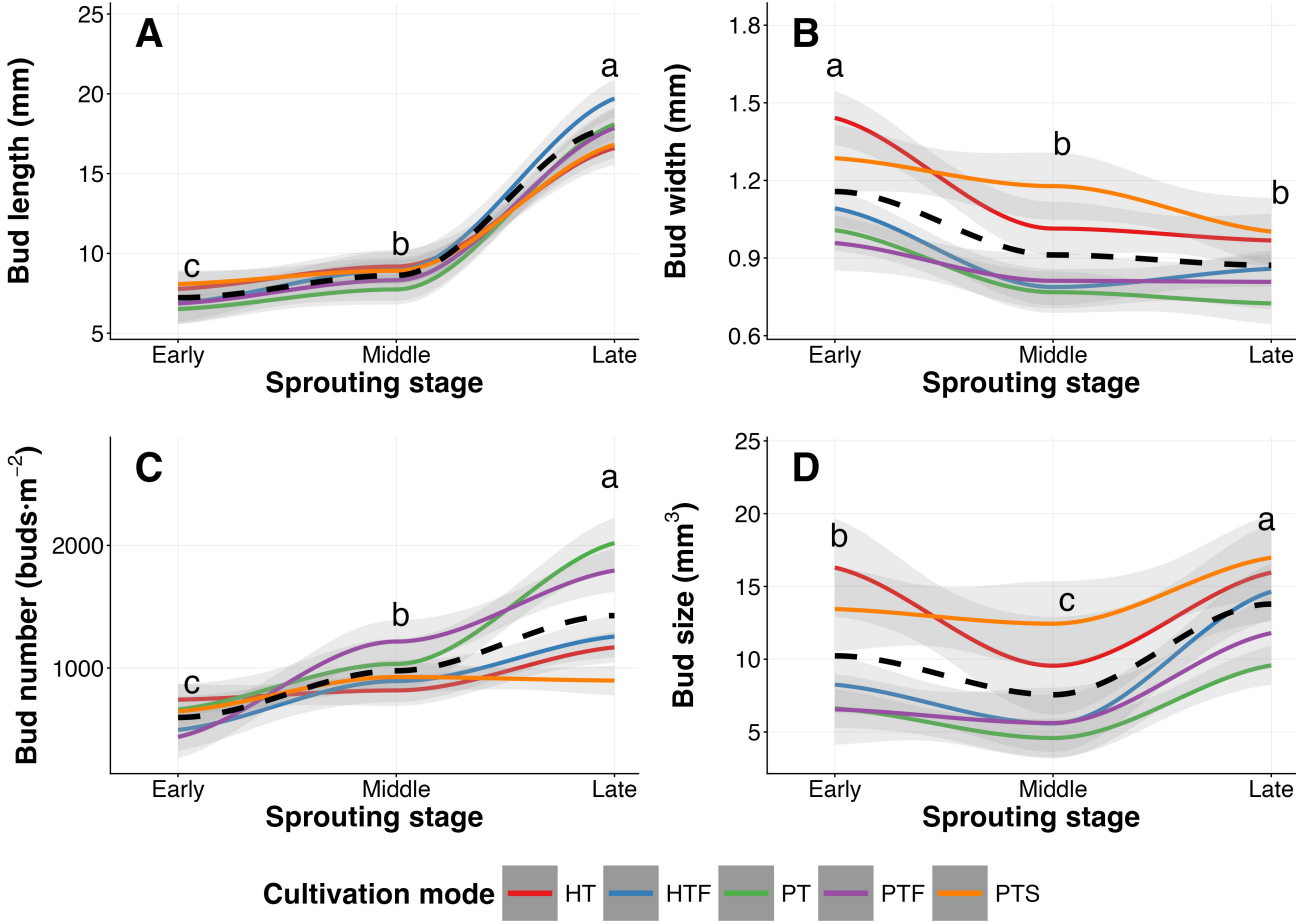
Dynamics of tea bud morphological traits across three sprouting stages under different cultivation modes (n = 5). Colored solid lines represent smoothed trends (loess fitting) for each cultivation mode; shaded areas indicate 95% confidence intervals. The black dashed line represents the overall mean trend across all cultivation modes. Different lowercase letters above the shaded areas indicate significant differences among stages based on Tukey’s HSD post−hoc tests (*P* < 0.05). Abbreviations for cultivation modes (HT, HTF, PT, PTF, PTS) are defined in **Table 1**.

The dynamic trends of various morphological indicators of tea buds were generally consistent across different cultivation modes (**Figure 1**). Tea bud length and number continuously increased throughout the sprouting process (*P* < 0.05; **Figures 1A, C**; **Supplementary Table S3**). The relative growth for bud length was 20% and 106% during the early-to-middle and middle-to-late stages, respectively. The corresponding relative growth for bud number were 74% and 44%. In contrast, bud width gradually decreased (**Figure 1B**; **Supplementary Table S3**), with a significant reduction of -21% during the early-to-middle stage (*P* < 0.05) and no significant change thereafter (*P* > 0.05). Bud size overall showed an initial decrease (relative change: -24%) followed by an increase (relative change: 92%) (*P* < 0.05; **Figure 1D**; **Supplementary Table S3**).

**Figure 2:**
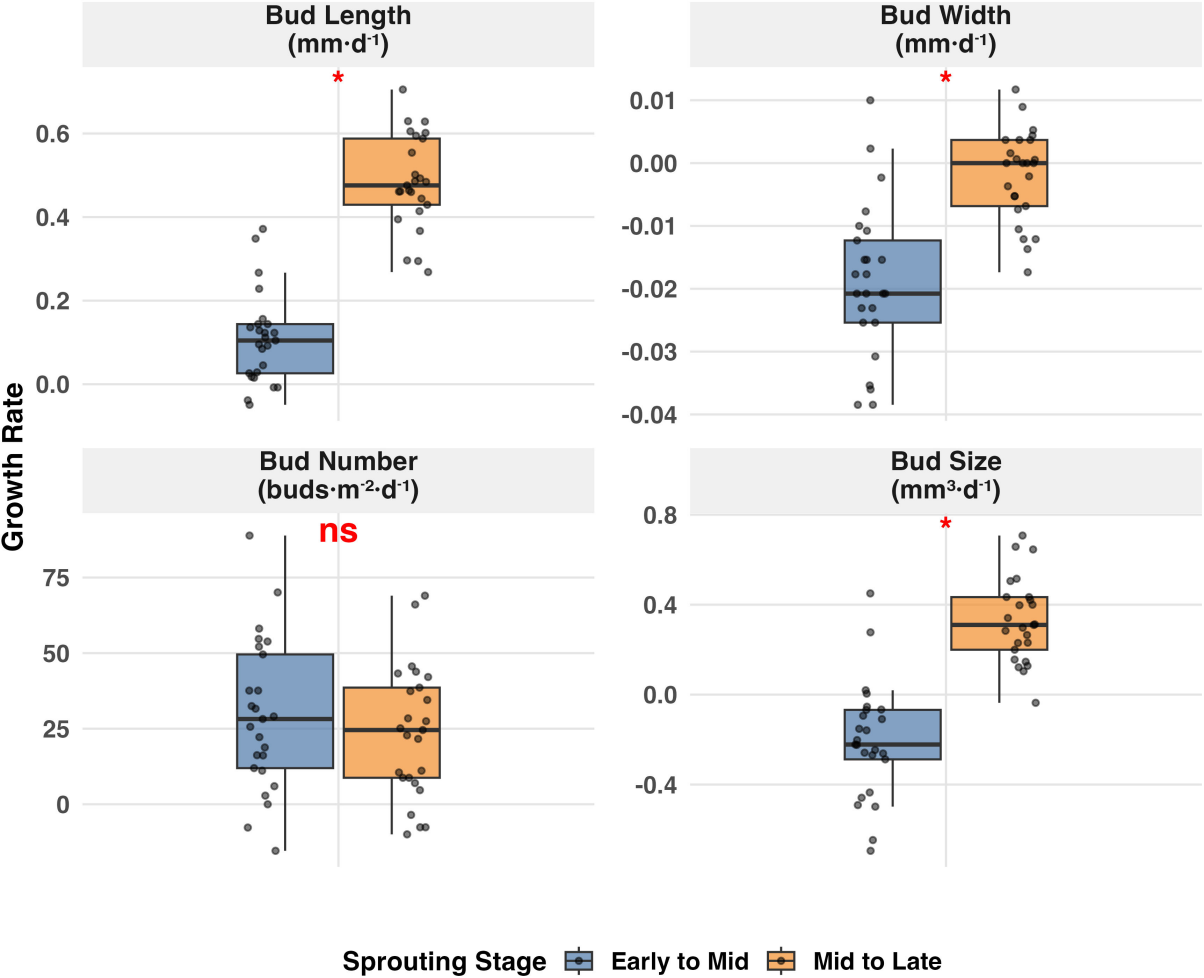
Comparison of daily growth rates of tea bud morphological traits between two successive sprouting periods across all cultivation modes (n = 25). Growth rates were calculated as the daily absolute change of each trait from early−to−mid (13 d) and mid−to−late (19 d) stages. Boxes show the interquartile range (IQR), horizontal lines denote medians, and points represent individual observations. Asterisks above the boxes indicate significance levels from paired−sample *t*-tests comparing the two periods (*, P < 0.05; ns, not significant).

Furthermore, the growth rates for tea bud length, width, and size during the middle-to-late stage were significantly higher than those during the early-to-middle stage (*P* < 0.05, **Figure 2**), whereas the growth rate for bud number did not differ significantly between the two stages (*P* > 0.05, **Figure 2**). This pattern was consistent across most cultivation modes (**Supplementary Figure S5**). Across all cultivation modes, the growth rates for length and number were consistently positive, whereas the rate for width remained negative. The growth rate for bud size shifted from negative to positive. These patterns of growth rates corroborate the consistent overall developmental trends described above (**Figure 1**; **Supplementary Figure S5**).

### 3.2 Differential effects of cultivation modes on tea bud morphology and chemical quality

**Figure 3:**
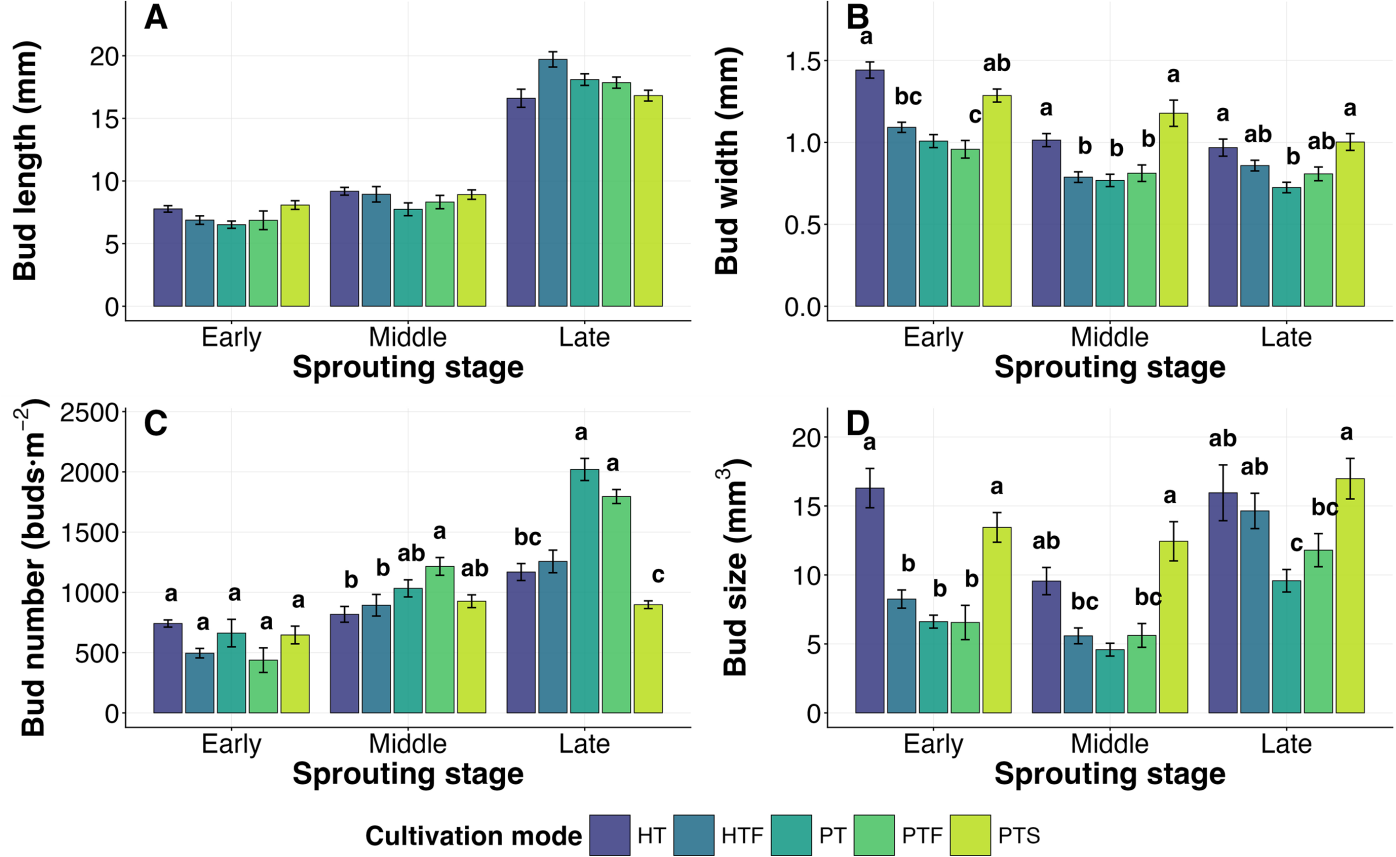
Effects of different cultivation modes on tea bud length, width, size, and number at different sprouting stages (n = 5). Different lowercase letters indicate significant differences among cultivation modes within the same sprouting stage according to Tukey’s HSD post−hoc test (*P* < 0.05) following linear mixed−effects models (fixed effect: cultivation × stage; random effect: quadrat). Absence of letters indicates no significant differences among modes at *P* > 0.05. Error bars represent standard errors of the mean. Cultivation mode abbreviations (HT, HTF, PT, PTF, PTS) are detailed in **Table 1**.

Cultivation mode had no significant effect on tea bud length at any of the three observed sprouting stages (*P* > 0.05; **Figure 3A**). In contrast, it exhibited a significant regulatory effect on bud width and size (*P* < 0.05; **Figures 3B, D**). Specifically, Hilly Tea Monoculture (HT) and Plain Tea-Soybean Intercropping (PTS) maintained larger bud width and size throughout the sprouting period, with their average values across stages being significantly higher by 24%–109% compared to other modes (*P* < 0.05; **Figures 3B, D**). The response of bud number to cultivation mode varied depending on the sprouting stage (**Figure 3C**). No cultivation mode showed a clear advantage at the early stage. However, by the middle and late stages, Plain Tea Monoculture (PT) and Plain Tea-Forest Intercropping (PTF) supported significantly higher densities than other modes, with this advantage further increasing to 72% at the late stage (*P* < 0.05; **Figure 3C**).

**Figure 4:**
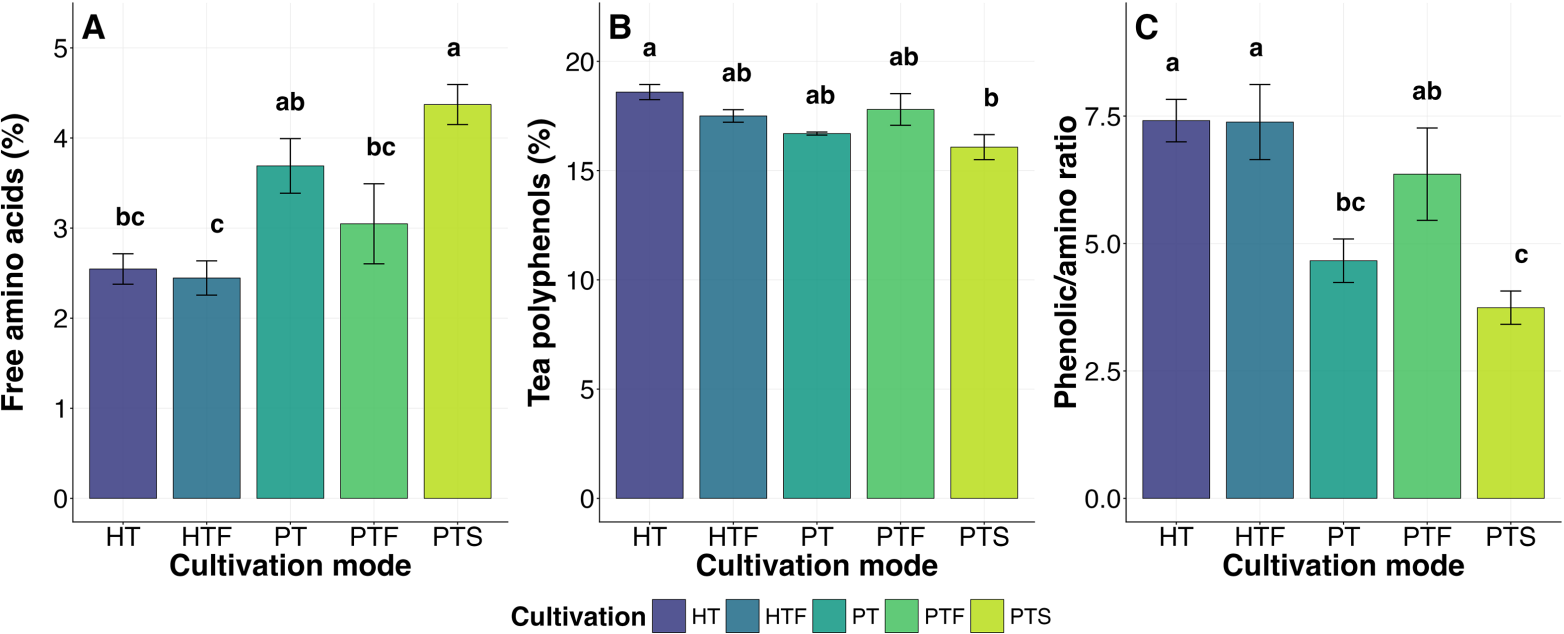
Effects of different cultivation modes on tea leaf chemical composition (n = 5). Different lowercase letters indicate significant differences among cultivation modes according to Tukey’s HSD post−hoc test (*P* < 0.05) following linear mixed−effects models (fixed effect: cultivation mode; random effect: quadrat). Error bars represent standard errors of the mean. Cultivation mode abbreviations (HT, HTF, PT, PTF, PTS) are defined in **Table 1**.

Regarding chemical quality, cultivation mode significantly affected all three chemical indicators (*P* < 0.05; **Figures 4A–C**). Specifically, PT and PTS were associated with a “refreshing” quality profile, characterized by the highest free amino acid content (4.0%; **Figure 4A**) and the lowest tea polyphenol content and polyphenol-to-amino acid ratio (16.4% and 4.26; **Figures 4B, C**). Conversely, HT and Hilly Tea-Forest Intercropping (HTF) were linked to a “mellow and robust” profile, exhibiting the lowest free amino acid content (2.5%; **Figure 4A**) and the highest tea polyphenol content and polyphenol-to-amino acid ratio (18.0% and 7.51; **Figures 4B, C**). The chemical indicators for PTF were intermediate between these two distinct categories.

### 3.3 Sprouting stage predominantly drove bud length, while cultivation mode more strongly influenced width and number

**Figure 5:**
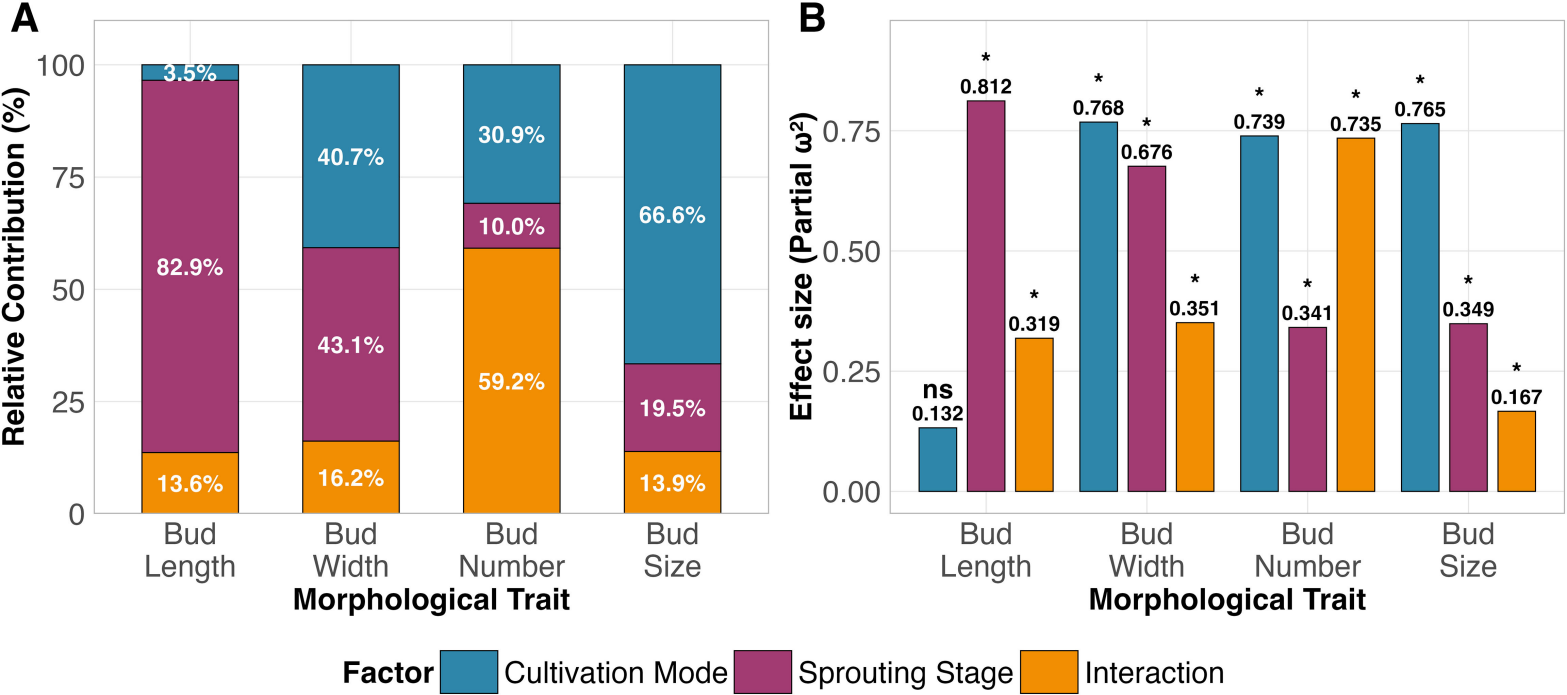
Contribution and effect size of cultivation mode, sprouting stage, and their interaction on tea bud morphological traits (n = 5). **(A)** Stacked bars show the relative contribution (%) of each factor to the total variation, calculated based on model III ANOVA from linear mixed−effects models (fixed effects: cultivation × stage; random effect: quadrat). **(B)** Grouped bars represent partial ω² effect sizes for each factor; asterisks denote that the factor had a significant effect on the corresponding indicator (*, *P* < 0.05; ns, not significant).

Except for its non-significant effect on bud length, cultivation mode, sprouting stage, and their interaction all had significant effects on the various tea bud morphological indicators (**Figure 5**; **Supplementary Table S3**). Specifically, the sprouting stage accounted for a substantially higher proportion of variance in bud length variation than cultivation mode did (82.9% *vs*. 3.5%; **Figure 5A**). Conversely, bud size was more sensitive to cultivation mode than to sprouting stage (66.6% *vs*. 19.5%; **Figure 5A**). The relative contributions of these two factors to bud width variation were similar (40.7% *vs*. 43.1%; **Figure 5A**). Notably, in the model for bud number, the interaction effect accounted for a greater proportion of variance (59.2%) than either factor alone (**Figure 5A**).

Effect size analysis revealed that cultivation mode had the smallest effect on bud length (partial ω² = 0.132), while its effects on the other three morphological indicators were similar (0.739–0.768) (**Figure 5B**). Sprouting stage had the largest effect on bud length (0.812), followed by its effect on bud width (0.676), and its effects on bud number and size were similar (0.341–0.349) (**Figure 5B**). The interaction effect was greatest on bud number (0.735), which was 2–4 times larger than its effects on the other three indicators (**Figure 5B**).

### 3.4 Cultivation mode had a stronger effect on morphological than on chemical quality traits

**Figure 6:**
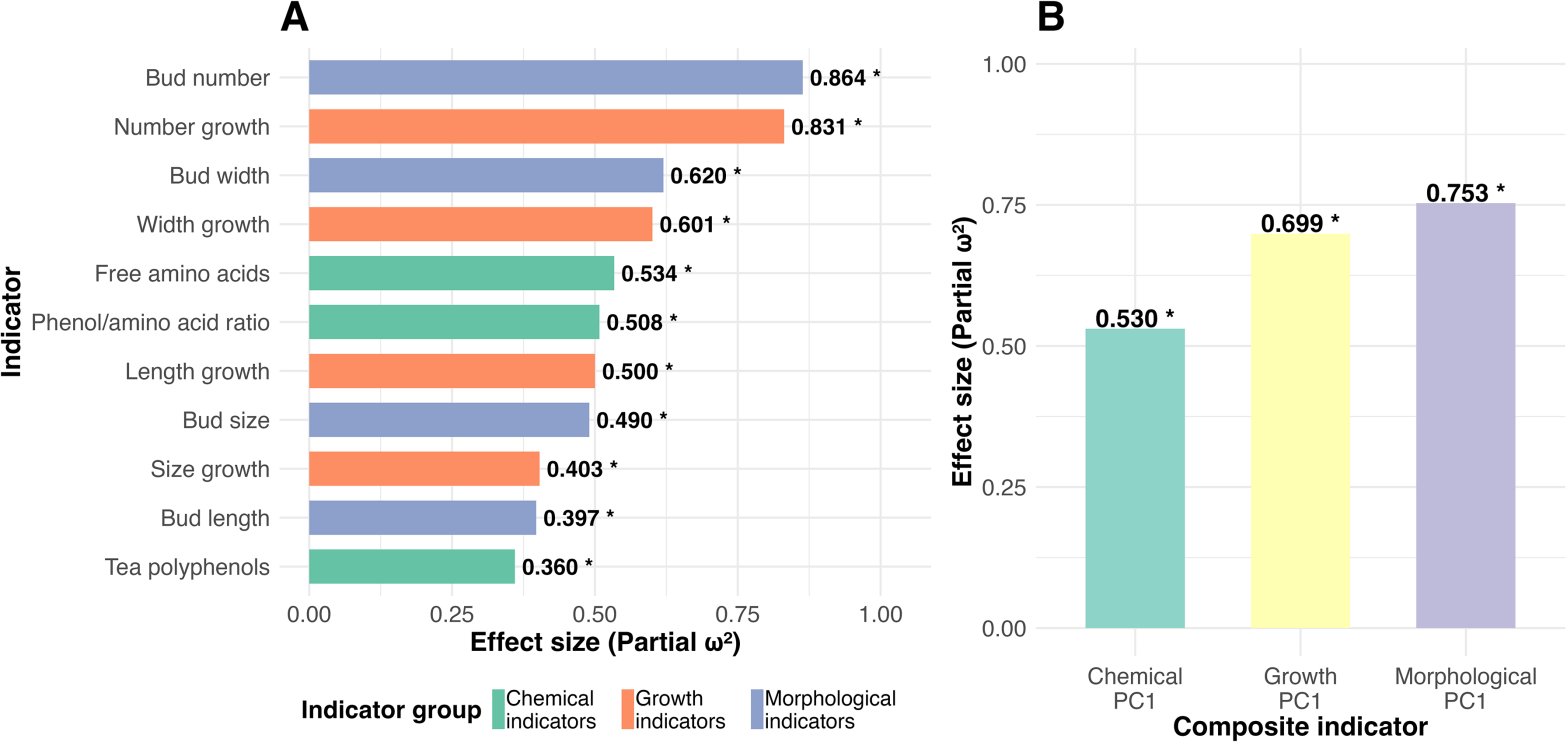
Effect sizes of cultivation modes on individual and composite indicators of tea chemistry, morphology, and growth (n = 5). **(A)** Partial ω² (partial omega−squared) effect sizes for individual indicators. **(B)** Partial ω² effect sizes for the first principal component (PC1) of each composite indicator group. Both panels present effect sizes estimated from separate linear mixed−effects models (fixed effect: cultivation mode; random effect: quadrat). Asterisks denote that the cultivation mode had a significant effect on the corresponding indicator (*, *P* < 0.05; ns, not significant).

Cultivation mode had significant effects on the chemical and morphological indicators measured at the late sprouting stage, as well as on the overall growth rates of tea buds during the entire sprouting period (**Figure 6**; **Supplementary Table S7**). Among these, cultivation mode exerted the strongest influence on late-stage bud number and its overall growth rate (partial ω²: 0.831–0.864), followed by late-stage bud width and its growth rate (0.601–0.620). Its effects on late-stage free amino acid content and the polyphenol-to-amino acid ratio ranked third (0.508–0.534) (**Figure 6A**). The effect of cultivation mode on the overall growth rate of length (0.500) was greater than its effects on late-stage bud size and its overall growth rate (0.403–0.490) (**Figure 6A**). Late-stage bud length and tea polyphenol content were the least responsive to cultivation mode (0.360–0.397) (**Figure 6A**). Overall, cultivation mode had a greater effect on the composite morphological index (0.753) than on the composite growth rate index (0.699) or the composite chemical quality index (0.530) (**Figure 6B**).

### 3.5 Correlations among morphological traits shifted dynamically across sprouting stages

**Figure 7:**
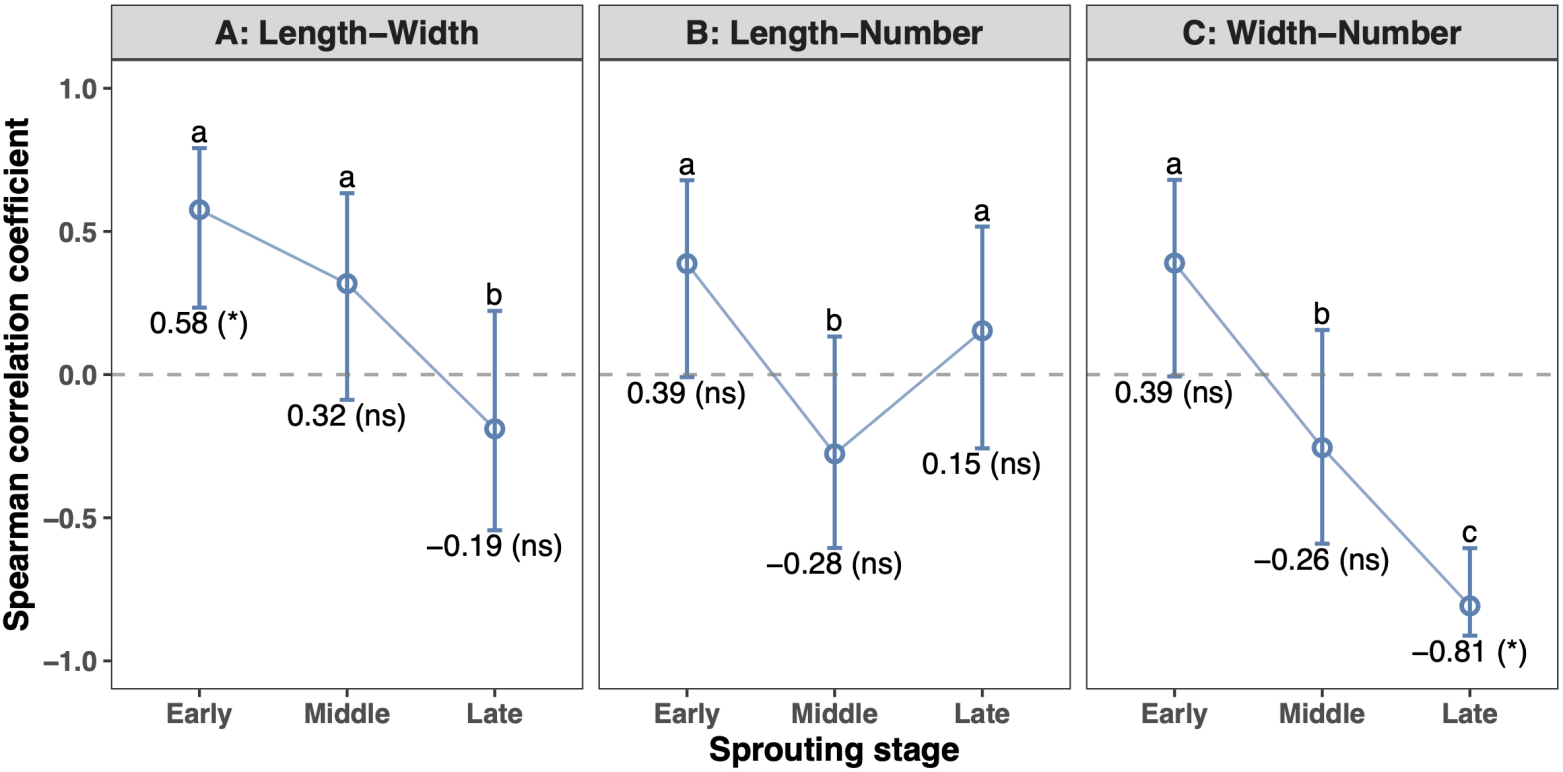
Changes in correlation between tea bud morphological traits across three sprouting stages (n = 5 per stage). Each panel shows the Spearman correlation coefficient (*r*_s_) for a specific trait pair. Points represent *r*_s_ values with 95% confidence intervals; asterisks indicate correlation significance (*, *P* < 0.05; ns, not significant). Different lowercase letters above points denote statistically significant differences between stages, as determined by non−parametric bootstrap permutation tests for the same trait pair. Horizontal dashed line denotes zero correlation.

Bud length and width were significantly positively correlated at the early sprouting stage (r = 0.58, *P* < 0.05) but showed no significant correlation at the middle or late stages (*P* > 0.05). The correlation coefficient between length and width shifted from positive to negative from the early to the late stage (*P* < 0.05; **Figure 7A**). Bud length and number were not significantly correlated at any of the three stages. Their correlation coefficient first decreased and then increased during sprouting, with the coefficients at the early and late stages both being significantly different from that at the middle stage (*P* < 0.05; **Figure 7B**). Bud width and number were significantly negatively correlated at the late stage (r = -0.81, *P* < 0.05), but not at the early or middle stages (*P* > 0.05). The correlation coefficient between width and number also declined from positive to negative from the early to the late stage (*P* < 0.05; **Figure 7C**).

### 3.6 Chemical quality was not significantly correlated with final bud morphology or growth rates

**Figure 8:**
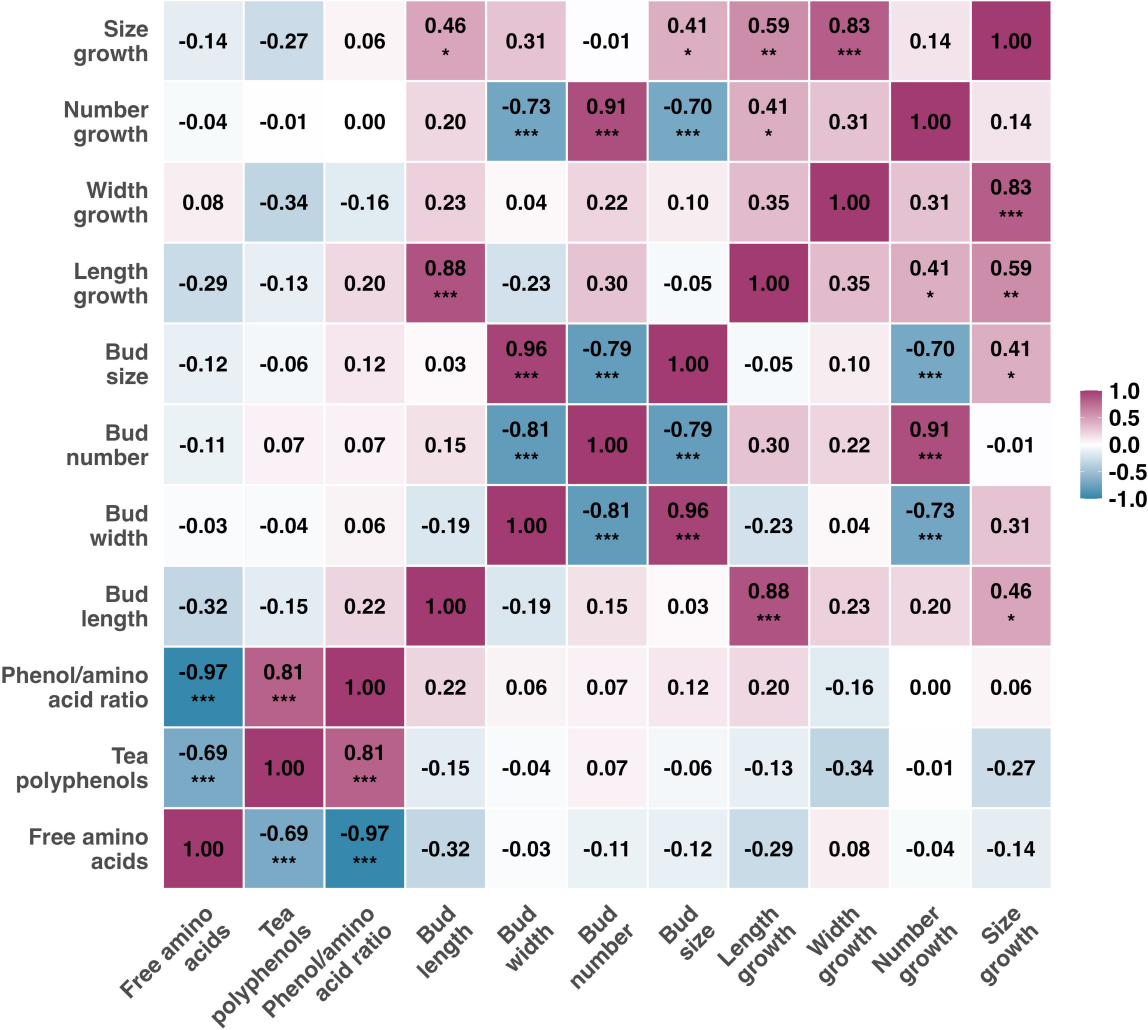
Spearman correlation analysis among chemical quality, final morphology, and growth rates (n = 5). Cells display correlation coefficients (*r*_s_) with significance stars (*, *P* < 0.05; **, *P* < 0.01; ***,*P* < 0.001; ns, not significant). Red and blue shades represent positive and negative correlations, respectively.

Systematic correlations were observed among the chemical indicators (**Figure 8**): free amino acids were significantly negatively correlated with tea polyphenols (r = -0.69;*P* < 0.05); the negative correlation between the polyphenol-to-amino acid ratio and free amino acids (r = -0.97;*P* < 0.05) was stronger than its positive correlation with tea polyphenols (r = 0.81;*P* < 0.05). Notably, no significant correlations were detected between any chemical indicators and the tea bud morphological indicators measured at the final stage or growth rates throughout the sprouting period (**Figure 8**). Further analysis revealed a positive correlation between the growth rates of bud number and length (r = 0.41; *P* < 0.05), while the growth rate of number was negatively correlated with both the late-stage bud width (r = -0.73; *P* < 0.05) and the growth rate of bud size (r = -0.70; *P* < 0.05). The growth rate of bud size was positively correlated with the growth rates of both length (r = 0.59; *P* < 0.05) and width (r = 0.83; *P* < 0.05) (**Figure 8**).

## 4 Discussion

### 4.1 Inherent program of bud morphological development and its phenotypic plasticity

As the primary harvesting target of premium teas, the dynamic morphological development of tea buds directly affects the yield of fresh leaves and is a crucial objective of tea garden agronomic management. Within the observational framework of three time points in this study, we found that despite the diversity in cultivation modes, the morphological development of tea buds exhibits an inherent conservatism: their length and number continuously increased throughout the sprouting period, width gradually decreased, while bud size showed an initial decrease followed by an increase. This overall trend remained consistent across different cultivation modes (**Figure 1**; **Supplementary Figure S5**). This consistent pattern indicates that tea bud development follows an inherent, genetically programmed blueprint (De Costa et al., 2007; Zhang et al., 2016, 2025b). However, in-depth analysis revealed significant differences in the responses of different morphological traits to external cultivation environments, uncovering the complex interaction between genetic control and agronomic plasticity.

Specifically, the development of tea bud length exhibited strong genetic constraint. This trait had an extremely high effect size for the sprouting stage (0.812) and a very low effect size for cultivation mode (0.132) (**Figure 5B**), indicating that its orderly elongation is a core event in the sprouting process, primarily driven by internal developmental signals, with limited scope for external cultivation regulation. In contrast, the development of tea bud width, size, and number showed higher phenotypic plasticity. Their response to cultivation mode was stronger than that of bud length (effect sizes 0.739—0.768, **Figure 4A**). Previous research has mostly focused on the genetic analysis of static morphology; for example, Zhang et al. (2025b) revealed key genes regulating tea bud size through digital phenotyping, GWAS, and transcriptomic analysis. Based on dynamic observations at limited time points, this study preliminarily outlines the plasticity patterns of tea bud morphological development, suggesting that width and number may be key traits responsive to cultivation regulation. This finding provides initial evidence and potential targets for considering tea bud width and number as key plastic traits for agronomic intervention, although the underlying patterns and mechanisms require further validation through higher-frequency continuous monitoring in the future.

During the early sprouting stage, tea bud length and width exhibited a significant positive correlation. Although the relationships between the other two pairs of morphological indicators did not reach statistical significance, they also showed positive associations (**Figures 7A–C**). This suggests that under conditions of sufficient resources in the initial sprouting phase, there is synergistic resource allocation among different morphological dimensions. As sprouting progressed, tea bud length and width, as well as width and number, showed negative correlations at the late sprouting stage, and these correlation coefficients were significantly different from those observed at the early stage (**Figures 7A, C**). This dynamic pattern clearly reveals a shift in tea bud developmental strategy: from synergistic growth in the early stage to a gradual shift towards competitive allocation of limited resources in the late stage between individual lateral expansion (width) and population density (number), as well as longitudinal extension (length). It is noteworthy that length and number showed no significant correlation throughout all periods (**Figure 7B**), while a significant positive correlation existed between the growth rates of length and number (**Figure 8**), suggesting a synergistic promotion between vertical elongation and population expansion. This finding aligns well with the classic plant size–density relationship theory in ecology and the dynamic optimization theory of plant growth (Callaway and Walker, 1997; Iwasa, 2000), and systematically verifies for the first time the existence of such a trade-off mechanism during tea bud sprouting. This intrinsic trade-off relationship provides a key mechanistic perspective for understanding the dynamic plasticity of morphological construction in tea plants.

Different cultivation modes possibly achieve differentiated stage-specific regulation of these plastic traits by altering the source-sink relationship and micro-environment of the tea plants. For instance, the Hilly Tea Monoculture (HT) and Plain Tea-Soybean Intercropping (PTS) modes maintained larger bud width and size throughout the sprouting period (**Figures 3B, D**). The width advantage in the HT mode might benefit from its relatively higher temperature and moderate radiation (**Table 1**), conditions considered conducive to promoting cell lateral expansion (Brown and Rickless, 1949; Casal and Balasubramanian, 2019). The advantage of the PTS mode likely stems from the unique biological nitrogen fixation of leguminous crops, providing a sufficient nitrogen nutrition base for the lateral growth of tea buds (Duan et al., 2021; Pokharel et al., 2023). On the other hand, the bud number advantage shown by Plain Tea Monoculture (PT) and Plain Tea-Forest Intercropping (PTF) in the mid-to-late stages (**Figure 3C**) might be related to the promotion of axillary bud differentiation by higher solar radiation in the PT mode (**Table 1** shows its highest solar radiation) (Leduc et al., 2014; Samuoliene et al., 2017).

### 4.2 Chemical quality divergence and potential micro-environmental drivers

Previous research generally holds that high-altitude environments, due to higher air humidity and diffuse light, usually favor increased free amino acid content, reduced tea polyphenol levels, and a lower polyphenol-to-amino acid ratio in tea, thereby forming a “refreshing” quality style (Han et al., 2017; Li et al., 2022; Ren et al., 2025). However, this study observed an opposite trend: plain tea gardens (PT/PTS) exhibited typical “refreshing” quality characteristics, while hilly tea gardens (HT/HTF) leaned towards a “mellow and robust” style. In this study, the core driver of chemical quality differentiation lies in the significant difference in free amino acid content, directly reflected in the significant change of the polyphenol-to-amino acid ratio (**Figures 4A, C**). In contrast, tea polyphenol content remained relatively stable across treatments (**Figures 4B**, **6A**), indicating that under the conditions of this study, the difference in tea chemical quality was primarily dominated by nitrogen metabolism products—especially the content of free amino acids—rather than the carbon metabolism product, tea polyphenols.

This free amino acid-dominated quality differentiation pattern profoundly reflects the differential regulation of tea plant nitrogen metabolism pathways by the micro-environments created by different cultivation modes. In the plain modes (PT/PTS), milder light, higher air humidity, and good soil moisture conditions (**Table 1**) collectively created a micro-environment favorable for nitrogen assimilation and amino acid accumulation (Jeyaramraja et al., 2003; Kc et al., 2021; Qifang et al., 2025). Among them, the PTS mode, further enhanced by the biological nitrogen fixation of leguminous crops, significantly increased rhizosphere nitrogen availability, strengthening its “refreshing” quality characteristics (Ruan et al., 2007; Duan et al., 2021; Wang et al., 2022). In contrast, amino acid accumulation was generally limited in the hilly modes (HT/HTF), but the limiting mechanisms differed: the lower soil water content (31.46%) in the HT mode might induce water stress, inhibiting the transport of amino acids from roots to new shoots (Good and Zaplachinski, 1994; Jeyaramraja et al., 2003; Qifang et al., 2025). Although the HTF mode had higher soil moisture and lower radiation, its lowest average temperature (16.69°C) was below the optimal growth temperature range for tea plants (20–25°C), significantly inhibiting the efficiency of theanine synthesis in roots and its transport to the apical parts (Wu et al., 2025), reflecting that water and temperature factors differentially dominated the suppression of nitrogen metabolism in different hilly modes.

This study further quantified, through effect sizes, that the regulatory effect of cultivation mode on the final morphology of tea buds monitored at the late sprouting stage (0.753) was greater than its effect on the simultaneously measured chemical quality indicators (0.530). Even the response of morphological growth rates (0.699) to cultivation mode was stronger than that of chemical quality (**Figure 6B**). This result clearly demonstrates that, on an agronomic timescale, the external morphological traits of tea buds exhibit higher cultivation controllability than their internal chemical quality traits.

To cope with unstable environments, plants can adapt by adjusting their morphological structure or by rapidly altering their metabolite composition (Dong and Lin, 2021; Zhang et al., 2022). However, these two adaptive strategies differ fundamentally in the underlying mechanisms of their plasticity. The production and turnover of secondary metabolites can be very rapid; their biochemical response to and cessation following environmental signals are far more sensitive and reversible than morphological adjustments (Metlen et al., 2009). In contrast, the morphogenesis of leaves or buds involves complex developmental programs such as cell division, expansion, and tissue differentiation. Changes in morphology are often irreversible or accumulate slowly (Fritz et al., 2018). Therefore, although metabolic traits are environmentally sensitive, their strong dynamic fluctuations make it difficult to achieve stable, directional, and cumulative changes through single or short-term agronomic practices. In contrast, once morphological traits undergo directional changes in response to environmental influences, they are more easily fixed and observed, thus exhibiting greater potential for agronomic manipulation.

This study is the first to quantitatively verify this principle within the tea plant system, revealing that the shaping effect of cultivation mode on morphology (particularly width and number) is significant and stable. Although environmental factors also drive changes in key tea metabolites (e.g., tea polyphenols, amino acids) (Wu et al., 2025), their responses are likely more susceptible to interference from multiple factors such as transient climatic fluctuations and developmental stage transitions. This may result in their effect size in response to cultivation mode being lower than that of morphological traits at a given point in time. This understanding not only deepens the comprehension of the hierarchical regulation of “morphology-metabolism” in tea garden ecosystems but also provides a key theoretical basis for prioritizing the directional optimization of tea bud appearance traits through precise microenvironment management (e.g., targeted regulation of light and temperature resources via intercropping or shading). Future research could further integrate continuous time-series dynamic monitoring to analyze the differential response trajectories of morphological and metabolic plasticity over longer cycles, thereby refining the process-based theoretical system for precision cultivation.

### 4.3 Stage-specific decoupling between morphology and chemistry

Through dynamic monitoring, this study found no significant correlations between all chemical quality indicators (free amino acids, tea polyphenols, and the polyphenol-to-amino acid ratio) and the morphological indicators of tea buds (length, width, size, number, and their growth rates) at the late sprouting stage (**Figure 8**). This clear “morphology-chemistry” decoupling phenomenon challenges the traditional empirical belief that intrinsic tea quality can be inferred from appearance traits, such as bud size. The underlying mechanism may stem from a fundamental hierarchical separation between the biological processes of morphogenesis and metabolite accumulation, in terms of their regulatory logic, temporal scales, and resource allocation.

From the perspective of molecular regulation, morphological development and chemical quality formation may be controlled by distinct genetic programs and environmental response networks. The morphogenesis of tea buds, especially key traits like size, is primarily governed by a relatively fixed developmental program. Research indicates that key genes such as CsKNOX6 play a central role in regulating tea bud cell division and expansion, and variation in their expression can significantly affect bud size, reflecting the strong genetic constraint on morphological development (Zhang et al., 2025b). More importantly, the morphological plasticity of leaves (buds) itself is a highly integrated, modular process controlled by complex gene regulatory networks (GRNs). Environmental signals (e.g., light, temperature) must influence key components within these networks, such as transcription factors, chromatin states, and hormone signaling pathways, to trigger amplified yet relatively slow morphological adjustments (Gonzalez et al., 2012; Fritz et al., 2018). This regulatory mode determines the “inertia” and cumulative effects of morphological traits in response to the environment.

In stark contrast, the biosynthesis and accumulation of key components determining tea flavor chemical quality—amino acids (e.g., theanine) and tea polyphenols (primarily catechins)—are governed by a highly dynamic and plastic metabolic network. These secondary metabolites are core components of the plant’s chemical defense system against environmental stresses (both biotic and abiotic), and their synthesis is characterized by rapid induction and reversibility (Metlen et al., 2009; Scheres and van der Putten, 2017). Substantial evidence shows that a range of environmental factors, including seasonal shifts, water stress, temperature fluctuations, and light changes, can rapidly and significantly (with variation amplitudes up to 50%) alter the content of secondary metabolites in tea leaves. However, the direction and extent of these effects vary depending on the specific environment, cultivar, and management practices, and there are even contradictory reports, highlighting the extreme complexity and context-dependency of metabolic network responses (Ahmed et al., 2019; Wu et al., 2025). In short, morphological development addresses “how to build a stable structure,” while chemical quality formation addresses “how to perform dynamic biochemical defense and adaptation.” There is an essential difference in their regulatory objectives and response dynamics.

This difference in regulatory hierarchy may lead to a temporal misalignment between the two processes on the developmental timescale. Morphological development (e.g., cell division, tissue differentiation) is a continuous, resource-intensive process, and its changes, once initiated, are not easily reversed. In contrast, the synthesis and turnover of secondary metabolites may fluctuate at a faster pace in response to transient environmental signals. The observations in this study were concentrated in the specific time window of the late sprouting stage, potentially capturing a “phase difference” between the key stage of morphological development and the dynamic equilibrium period of chemical substances. This temporal asynchrony might be an important reason for observing phenotypic decoupling at a single time point. Other studies also hint at the complexity of morphology-metabolism relationships. For instance, in tea leaves, photosynthetic and respiratory parameters were significantly correlated with tea polyphenol content but not with total amino acid content, indicating that the coupling strength between different metabolic pathways and physiological functions inherently differs (Li et al., 2016).

Furthermore, internal resource allocation trade-offs within the plant may provide a further explanation for the observed stage-specific decoupling phenomenon. The lack of correlation between tea bud morphogenesis (growth) and chemical quality formation (defense) can be interpreted through the classic “ theory of plant defense” in plant ecology. This theory posits that under limited resource conditions, plants cannot simultaneously maximize investment in both primary growth (e.g., tissue expansion) and secondary metabolic defense (e.g., synthesis of phenolic compounds) (Coley et al., 1985; Herms and Mattson, 1992; Stamp, 2003). As mentioned above, our study indicates that trade-offs already exist among different morphological components during plant growth by the late sprouting stage (e.g., between bud “width” and “number”) (**Figure 7**). Therefore, during the rapid sprouting phase, the developmental program likely prioritizes the allocation of resources to core morphogenetic processes, while the allocation for synthesizing key secondary metabolites (e.g., theanine, catechins) is relatively constrained. This differential resource allocation at critical developmental stages can lead to independent variation trajectories between morphological and chemical traits—that is, the observed stage-specific decoupling phenomenon. In summary, this suggests that morphological construction and chemical quality formation may operate as partially independent modules. Consequently, agronomic measures targeting one module may not induce proportional or linear changes in the other.

The stage-specific decoupling between morphology and chemistry implies that yield- and quality-related traits in tea may be regulated independently. However, this study’s sampling of chemical quality only at a single, late developmental stage limits our ability to characterize dynamic relationships or transient coordination between these trait categories across the entire sprouting period. Therefore, future research should integrate time−series multi−omics (transcriptomics, metabolomics) with continuous, precise phenotyping to dynamically track the trajectories of morphological and chemical traits and to identify the key regulatory nodes underlying their independent or coupled expression.

### 4.4 Implications for precision cultivation practices

The systematic understanding described above lays an important foundation for developing quality-oriented tea garden management strategies. The results of this study can provide references for integrating multi-level strategies from macro-mode selection to field micro-management. The following preliminary ideas are elaborated to offer insights for the targeted regulation of tea quality.

First, in the strategic selection of cultivation modes, it should be closely aligned with the quality requirements of the target tea category. The results of this study indicate that Plain Tea-Soybean Intercropping (PTS) is the preferred mode for producing “refreshing” premium green tea, as it combines the highest free amino acid content (4.3%) with significantly larger tea bud width (**Figures 3B**, **4A**), providing ideal raw material for shaping high-end green tea with “good taste and beautiful appearance”. Conversely, Hilly Tea Monoculture (HT), due to its higher polyphenol-to-amino acid ratio and maintained good bud width (**Figures 3B**, **4A**), is more suitable for developing “mellow and robust” black tea products. This quality potential-based mode matching is a key decision for maximizing tea garden ecosystem services and economic benefits at the source.

Secondly, during the dynamic process of tea bud sprouting, stage-specific precise morphological regulation should be implemented. Based on the inherent programmability and plasticity windows of tea bud development, management measures need to vary with time. In the early sprouting stage, when tea bud width shows a significant decrease (-21%) (**Figure 1B**), the management core should focus on “preserving width”, potentially through strategies like foliar application of calcium fertilizer to enhance cell wall stability and delay lateral contraction. Entering the mid-to-late sprouting stage, when the increase in tea bud length significantly accelerates and the growth rate of tea bud number decreases (**Figures 1A, C**), the focus should shift to “promoting length and stabilizing density”, potentially by ensuring timely nitrogen supply to promote longitudinal elongation.

Finally, in response to the stage-specific “morphology-chemistry” decoupling phenomenon, a new paradigm of synergistic management must be developed. We propose establishing a “dual-track monitoring” system to achieve parallel optimization of yield and quality. Track one: use image analysis technology to track key morphological indicators (e.g., width/number ratio) in real-time (Zhang et al., 2019; Jiang et al., 2025a; Sun et al., 2025). When the absolute value of their correlation (|r|) exceeds the competition threshold of 0.5, guide corresponding “width-preserving” or “density-increasing” agronomic operations promptly. Track two, which is key to breaking the decoupling bottleneck, is to actively explore the use of multispectral or hyperspectral remote sensing technology to directly and non-destructively monitor the chemical components of young shoots (e.g., amino acid accumulation dynamics) (Ren et al., 2024; Jiang et al., 2025b). This would bypass the traditional misconception of indirectly inferring quality from morphology, ultimately enabling direct and precise regulation of tea chemical quality.

## 5 Conclusions

This study demonstrates that tea bud development follows a conserved program yet exhibits distinct trait-specific plasticity. While bud length was strongly governed by the sprouting stage, width, and number were more responsive to cultivation practices. Morphological traits were significantly more malleable to cultivation mode than chemical quality. Furthermore, the correlations among morphological traits shifted dynamically during sprouting: bud length and width transitioned from positive to negative correlation, as did width and number, indicating a progressive shift in resource allocation priorities during development.

Notably, at the final sprouting stage measured in this study, no significant correlations were detected between morphological traits and chemical quality indicators. This observed absence of correlation is based on analysis from a single, late developmental time point, which may not capture potential transient or stage-specific relationships that could occur earlier in bud development. Nevertheless, the clear lack of association at this key harvest-relevant stage challenges the widespread traditional assumption that larger buds inherently indicate better tea quality. Further research employing continuous temporal monitoring of both morphological and chemical dynamics will be important to fully clarify their relationship across the entire developmental progression.

Collectively, this work advances our understanding of gene-environment interactions in perennial crops and provides an actionable framework for precision management to simultaneously optimize tea yield and quality.

## Data Availability

The raw data supporting the conclusions of this article will be made available by the authors, without undue reservation.
