# Supplementary material for: Quantifying the effects of cultivation mode and sprouting stage on tea bud morphology and chemical quality
Source: Front Plant Sci. 2026 Jan 30;17:1747538. doi: 10.3389/fpls.2026.1747538 (PMC12903278; doi:10.3389/fpls.2026.1747538)
Supplement: Supplementary file 1 [file DataSheet1.docx]

Supplementary Materials: Complete Model Formulations, Figures, and Tables

Table of Contents

[Supplementary Materials 1](#_Toc218468125)

[1. Model Formulations 1](#_Toc218468126)

[1.1 Independent Structure Model (INDEP) 1](#_Toc218468127)

[1.2 Compound Symmetry Model (CS) 2](#_Toc218468128)

[1.3 First-order Autoregressive Model (AR1) 2](#_Toc218468129)

[1.4 Heterogeneous Compound Symmetry Model (CSH) 2](#_Toc218468130)

[1.5 Toeplitz Structure Model (TOEP) 3](#_Toc218468131)

[1.6 Unstructured Model (UN) 3](#_Toc218468132)

[2. Supplementary Tables and Figures 3](#_Toc218468133)

[2.1 Part I: Effects of Cultivation Mode and Sprouting Stage on Tea Bud Morphology and Growth Rates 3](#_Toc218468134)

[2.2 Part II: Effects of Cultivation Modes on Chemical Quality, Final Morphology, and Growth Rates 15](#_Toc218468135)

[2.3 Part III: Correlation Analysis Among Indicators 34](#_Toc218468136)

# Supplementary Materials

## 1. Model Formulations

The following six residual covariance structures were systematically compared for repeated-measures analysis of tea bud morphological traits.

### 1.1 Independent Structure Model (INDEP)

The model is formulated as:

$$Y_{ijk}=\mu+\alpha_{i}+\beta_{j}+\left( \alpha\beta\right)_{ij}+b_{k}+\epsilon_{ijk}$$

where: - $Y_{ijk}$: Observed value for cultivation mode $i$, sprouting stage $j$, and replicate $k$ - $\mu$: Overall mean - $\alpha_{i}$: Fixed effect of cultivation mode $i$ ($i=1,...,5$) - $\beta_{j}$: Fixed effect of sprouting stage $j$ ($j=1,2,3$) - $\left( \alpha\beta\right)_{ij}$: Interaction effect between cultivation mode and sprouting stage - $b_{k}\sim N\left( 0,\sigma_{b}^{2} \right)$: Random intercept for quadrat $k$ ($k=1,...,25$) - $\epsilon_{ijk}\sim N\left( 0,\sigma^{2} \right)$: Independent and identically distributed residuals

**Covariance structure:** $\text{Cov}\left( \epsilon_{ijk},\epsilon_{ij'k} \right)=0$ (independent measurements across time points)

### 1.2 Compound Symmetry Model (CS)

The model structure is identical to INDEP:

$$Y_{ijk}=\mu+\alpha_{i}+\beta_{j}+\left( \alpha\beta\right)_{ij}+b_{k}+\epsilon_{ijk}$$

**With residual covariance matrix:**

$$\left[ \begin{matrix} \epsilon_{i1k} \\ \epsilon_{i2k} \\ \epsilon_{i3k} \end{matrix} \right]\sim N\left( \left[ \begin{matrix} 0 \\ 0 \\ 0 \end{matrix} \right],\left[ \begin{matrix} \sigma^{2} & \rho\sigma^{2} & \rho\sigma^{2} \\ \rho\sigma^{2} & \sigma^{2} & \rho\sigma^{2} \\ \rho\sigma^{2} & \rho\sigma^{2} & \sigma^{2} \end{matrix} \right] \right)$$

**Where** $\rho$ **is the intra-class correlation coefficient.**

**Covariance structure:** Equal covariance between all time points, $\text{Cov}\left( \epsilon_{ijk},\epsilon_{ij'k} \right)=\rho\sigma^{2}$

### 1.3 First-order Autoregressive Model (AR1)

$$Y_{ijk}=\mu+\alpha_{i}+\beta_{j}+\left( \alpha\beta\right)_{ij}+b_{k}+\epsilon_{ijk}$$

**With residual covariance matrix:**

$$\Sigma=\sigma^{2}\left[ \begin{matrix} 1 & \rho& \rho^{2} \\ \rho& 1 & \rho\\ \rho^{2} & \rho& 1 \end{matrix} \right]$$

**Covariance structure:** $\text{Cov}\left( \epsilon_{ijk},\epsilon_{ij'k} \right)=\sigma^{2}\rho^{\left| j-j' \right|}$, with correlation decaying exponentially with time interval

### 1.4 Heterogeneous Compound Symmetry Model (CSH)

$$Y_{ijk}=\mu+\alpha_{i}+\beta_{j}+\left( \alpha\beta\right)_{ij}+b_{k}+\epsilon_{ijk}$$

**With residual covariance matrix:**

$$\Sigma=\left[ \begin{matrix} \sigma_{1}^{2} & \rho\sigma_{1}\sigma_{2} & \rho\sigma_{1}\sigma_{3} \\ \rho\sigma_{1}\sigma_{2} & \sigma_{2}^{2} & \rho\sigma_{2}\sigma_{3} \\ \rho\sigma_{1}\sigma_{3} & \rho\sigma_{2}\sigma_{3} & \sigma_{3}^{2} \end{matrix} \right]$$

**Covariance structure:** Equal within-group correlations but unequal variances, $\text{Var}\left( \epsilon_{ijk} \right)=\sigma_{j}^{2}$, $\text{Cov}\left( \epsilon_{ijk},\epsilon_{ij'k} \right)=\rho\sigma_{j}\sigma_{j'}$

### 1.5 Toeplitz Structure Model (TOEP)

$$Y_{ijk}=\mu+\alpha_{i}+\beta_{j}+\left( \alpha\beta\right)_{ij}+b_{k}+\epsilon_{ijk}$$

**With residual covariance matrix:**

$$\Sigma=\left[ \begin{matrix} \sigma^{2} & \sigma_{1} & \sigma_{2} \\ \sigma_{1} & \sigma^{2} & \sigma_{1} \\ \sigma_{2} & \sigma_{1} & \sigma^{2} \end{matrix} \right]$$

**Covariance structure:** $\text{Cov}\left( \epsilon_{ijk},\epsilon_{ij'k} \right)=\sigma_{\left| j-j' \right|}$, depending only on the time interval

### 1.6 Unstructured Model (UN)

$$Y_{ijk}=\mu+\alpha_{i}+\beta_{j}+\left( \alpha\beta\right)_{ij}+b_{k}+\epsilon_{ijk}$$

**With residual covariance matrix:**

$$\Sigma=\left[ \begin{matrix} \sigma_{1}^{2} & \sigma_{12} & \sigma_{13} \\ \sigma_{12} & \sigma_{2}^{2} & \sigma_{23} \\ \sigma_{13} & \sigma_{23} & \sigma_{3}^{2} \end{matrix} \right]$$

**Covariance structure:** No specific pattern, each variance and covariance estimated independently, $\text{Cov}\left( \epsilon_{ijk},\epsilon_{ij'k} \right)=\sigma_{jj'}$

## 2. Supplementary Tables and Figures

### 2.1 Part I: Effects of Cultivation Mode and Sprouting Stage on Tea Bud Morphology and Growth Rates

#### 2.1.1 Model Selection Results

**Table S1.** Model comparison for tea bud morphological traits based on Akaike Information Criterion (AIC), Bayesian Information Criterion (BIC), and AIC weights. Six covariance structures were tested: Independent (INDEP), Compound Symmetry (CS), First-order Autoregressive (AR1), Heterogeneous Compound Symmetry (CSH), Toeplitz (TOEP), and Unstructured (UN).

| Indicators | Model | AIC | BIC | logLik | df | deltaAIC | AIC weights |
| --- | --- | --- | --- | --- | --- | --- | --- |
| Bud length | INDEP | 244.80 | 280.40 | -105.40 | 17 | 0 | 0.37 |
|  | CS | 245.62 | 283.32 | -104.81 | 18 | 0.82 | 0.24 |
|  | AR1 | 246.10 | 283.80 | -105.05 | 18 | 1.30 | 0.19 |
|  | TOEP | 247.62 | 287.42 | -104.81 | 19 | 2.82 | 0.09 |
|  | CSH | 248.44 | 290.32 | -104.22 | 20 | 3.64 | 0.06 |
|  | UN | 249.47 | 291.36 | -104.73 | 20 | 4.67 | 0.03 |
|  |  |  |  |  |  |  |  |
| Bud width | INDEP | -43.87 | -8.266 | 38.9352 | 17 | 0 | 0.42 |
|  | AR1 | -42.96 | -5.270 | 39.4842 | 18 | 0.90 | 0.27 |
|  | CS | -41.87 | -4.172 | 38.9352 | 18 | 1.99 | 0.15 |
|  | TOEP | -40.97 | -1.181 | 39.4871 | 19 | 2.89 | 0.10 |
|  | UN | -39.05 | 2.8348 | 39.5260 | 20 | 4.81 | 0.03 |
|  |  |  |  |  |  |  |  |
| Bud number | AR1 | 847.37 | 885.07 | -405.68 | 18 | 0 | 0.37 |
|  | INDEP | 847.88 | 883.48 | -406.94 | 17 | 0.50 | 0.29 |
|  | TOEP | 849.37 | 889.17 | -405.68 | 19 | 2.00 | 0.13 |
|  | CS | 849.87 | 887.57 | -406.93 | 18 | 2.49 | 0.10 |
|  | UN | 850.86 | 892.75 | -405.43 | 20 | 3.48 | 0.06 |
|  | CSH | 853.62 | 895.51 | -406.81 | 20 | 6.25 | 0.01 |
|  |  |  |  |  |  |  |  |
| Bud size | INDEP | 345.87 | 381.48 | -155.93 | 17 | 0 | 0.38 |
|  | CSH | 347.31 | 389.20 | -153.65 | 20 | 1.43 | 0.18 |
|  | AR1 | 347.34 | 385.04 | -155.67 | 18 | 1.47 | 0.18 |
|  | CS | 347.87 | 385.57 | -155.93 | 18 | 1.99 | 0.14 |
|  | TOEP | 349.34 | 389.13 | -155.67 | 19 | 3.46 | 0.06 |
|  | UN | 351.16 | 393.05 | -155.58 | 20 | 5.28 | 0.02 |

Note: The CSH model was not included in the model selection for bud width because it failed to converge.

#### 2.1.2 Model Diagnostics

**Table S2.** Comprehensive diagnostic tests for the best-fitted models of four tea bud morphological traits (bud length, width, number, and size). Tests include Shapiro-Wilk normality test, skewness, kurtosis, Levene’s homogeneity of variance test, Ljung-Box autocorrelation test, and R-squared values (marginal and conditional).

| Variable | Model | Normality_Shapiro_W | Normality_Shapiro_p | Normality_Conclusion | Skewness | Kurtosis | Homogeneity_Levene_p | Homogeneity_Conclusion | Autocorrelation_ACF | Autocorrelation_LB_p | Marginal_R2 | Conditional_R2 |
| --- | --- | --- | --- | --- | --- | --- | --- | --- | --- | --- | --- | --- |
| Bud length | INDEP | 0.985 | 0.549 | Normal | -0.215 | 2.685 | 0.770 | Homogeneous | -0.141 | 0.213 | 0.950 | 0.950 |
| Bud width | INDEP | 0.962 | 0.023 | Not Normal | -0.356 | 2.282 | 0.371 | Homogeneous | -0.087 | 0.441 | 0.788 | 0.851 |
| Bud number | AR1 | 0.994 | 0.987 | Normal | 0.056 | 2.630 | 0.858 | Homogeneous | 0.037 | 0.742 | 0.870 | 0.890 |
| Bud size | INDEP | 0.971 | 0.087 | Normal | -0.602 | 3.318 | 0.090 | Homogeneous | -0.189 | 0.095 | 0.726 | 0.759 |

**Table S3.** Type III analysis of variance (ANOVA) table for the best-fitted mixed-effects models of four tea bud morphological traits, showing the effects of cultivation mode, sprouting stage, and their interaction.

|  |  | numDF | denDF | F-value | p-value |
| --- | --- | --- | --- | --- | --- |
| Bud length | (Intercept) | 1 | 40 | 1564.90 | 0.000 |
|  | Cultivation | 4 | 20 | 1.95 | 0.141 |
|  | Stage | 2 | 40 | 93.95 | 0.000 |
|  | Cultivation:Stage | 8 | 40 | 3.86 | 0.002 |
|  |  |  |  |  |  |
| Bud width | (Intercept) | 1 | 40 | 1161.56 | 0.000 |
|  | Cultivation | 4 | 20 | 21.69 | 0.000 |
|  | Stage | 2 | 40 | 45.89 | 0.000 |
|  | Cultivation:Stage | 8 | 40 | 4.31 | 0.001 |
|  |  |  |  |  |  |
| Bud number | (Intercept) | 1 | 40 | 467.37 | 0.000 |
|  | Cultivation | 4 | 20 | 18.72 | 0.000 |
|  | Stage | 2 | 40 | 12.12 | 0.000 |
|  | Cultivation:Stage | 8 | 40 | 17.95 | 0.000 |
|  |  |  |  |  |  |
| Bud size | (Intercept) | 1 | 40 | 358.32 | 0.000 |
|  | Cultivation | 4 | 20 | 21.33 | 0.000 |
|  | Stage | 2 | 40 | 12.51 | 0.000 |
|  | Cultivation:Stage | 8 | 40 | 2.22 | 0.046 |

**Table S4.** Fixed effects coefficients table for the best-fitted mixed-effects models, including estimates, standard errors, degrees of freedom, t-values, and p-values for each predictor.

|  |  | Value | Std.Error | DF | t-value | p-value |
| --- | --- | --- | --- | --- | --- | --- |
| Bud length | (Intercept) | 11.186 | 0.283 | 40 | 39.559 | 0.000 |
|  | CultivationHTF | 0.656 | 0.400 | 20 | 1.641 | 0.116 |
|  | CultivationPT | -0.402 | 0.400 | 20 | -1.006 | 0.326 |
|  | CultivationPTF | -0.174 | 0.400 | 20 | -0.435 | 0.669 |
|  | CultivationPTS | 0.084 | 0.400 | 20 | 0.211 | 0.835 |
|  | Stage.L | 6.247 | 0.490 | 40 | 12.757 | 0.000 |
|  | Stage.Q | 2.457 | 0.490 | 40 | 5.017 | 0.000 |
|  | CultivationHTF:Stage.L | 2.827 | 0.693 | 40 | 4.082 | 0.000 |
|  | CultivationPT:Stage.L | 1.945 | 0.693 | 40 | 2.808 | 0.008 |
|  | CultivationPTF:Stage.L | 1.526 | 0.693 | 40 | 2.203 | 0.033 |
|  | CultivationPTS:Stage.L | -0.069 | 0.693 | 40 | -0.100 | 0.921 |
|  | CultivationHTF:Stage.Q | 1.100 | 0.693 | 40 | 1.588 | 0.120 |
|  | CultivationPT:Stage.Q | 1.268 | 0.693 | 40 | 1.832 | 0.074 |
|  | CultivationPTF:Stage.Q | 0.840 | 0.693 | 40 | 1.214 | 0.232 |
|  | CultivationPTS:Stage.Q | 0.429 | 0.693 | 40 | 0.619 | 0.539 |
|  |  |  |  |  |  |  |
| Bud width | (Intercept) | 1.141 | 0.033 | 40 | 34.082 | 0.000 |
|  | CultivationHTF | -0.228 | 0.047 | 20 | -4.823 | 0.000 |
|  | CultivationPT | -0.308 | 0.047 | 20 | -6.497 | 0.000 |
|  | CultivationPTF | -0.282 | 0.047 | 20 | -5.953 | 0.000 |
|  | CultivationPTS | 0.014 | 0.047 | 20 | 0.301 | 0.766 |
|  | Stage.L | -0.335 | 0.039 | 40 | -8.686 | 0.000 |
|  | Stage.Q | 0.156 | 0.039 | 40 | 4.041 | 0.000 |
|  | CultivationHTF:Stage.L | 0.170 | 0.055 | 40 | 3.114 | 0.003 |
|  | CultivationPT:Stage.L | 0.135 | 0.055 | 40 | 2.468 | 0.018 |
|  | CultivationPTF:Stage.L | 0.229 | 0.055 | 40 | 4.197 | 0.000 |
|  | CultivationPTS:Stage.L | 0.134 | 0.055 | 40 | 2.465 | 0.018 |
|  | CultivationHTF:Stage.Q | -0.003 | 0.055 | 40 | -0.054 | 0.958 |
|  | CultivationPT:Stage.Q | -0.076 | 0.055 | 40 | -1.385 | 0.174 |
|  | CultivationPTF:Stage.Q | -0.098 | 0.055 | 40 | -1.794 | 0.080 |
|  | CultivationPTS:Stage.Q | -0.183 | 0.055 | 40 | -3.363 | 0.002 |
|  |  |  |  |  |  |  |
| Bud number | (Intercept) | 909.444 | 42.067 | 40 | 21.619 | 0.000 |
|  | CultivationHTF | -27.519 | 59.492 | 20 | -0.463 | 0.649 |
|  | CultivationPT | 329.074 | 59.492 | 20 | 5.531 | 0.000 |
|  | CultivationPTF | 240.185 | 59.492 | 20 | 4.037 | 0.001 |
|  | CultivationPTS | -85.741 | 59.492 | 20 | -1.441 | 0.165 |
|  | Stage.L | 302.092 | 63.657 | 40 | 4.746 | 0.000 |
|  | Stage.Q | 112.268 | 85.444 | 40 | 1.314 | 0.196 |
|  | CultivationHTF:Stage.L | 236.252 | 90.025 | 40 | 2.624 | 0.012 |
|  | CultivationPT:Stage.L | 658.002 | 90.025 | 40 | 7.309 | 0.000 |
|  | CultivationPTF:Stage.L | 658.002 | 90.025 | 40 | 7.309 | 0.000 |
|  | CultivationPTS:Stage.L | -124.529 | 90.025 | 40 | -1.383 | 0.174 |
|  | CultivationHTF:Stage.Q | -126.239 | 120.836 | 40 | -1.045 | 0.302 |
|  | CultivationPT:Stage.Q | 139.031 | 120.836 | 40 | 1.151 | 0.257 |
|  | CultivationPTF:Stage.Q | -193.011 | 120.836 | 40 | -1.597 | 0.118 |
|  | CultivationPTS:Stage.Q | -238.372 | 120.836 | 40 | -1.973 | 0.055 |
|  |  |  |  |  |  |  |
| Bud size | (Intercept) | 13.935 | 0.736 | 40 | 18.929 | 0.000 |
|  | CultivationHTF | -4.442 | 1.041 | 20 | -4.267 | 0.000 |
|  | CultivationPT | -7.009 | 1.041 | 20 | -6.732 | 0.000 |
|  | CultivationPTF | -5.946 | 1.041 | 20 | -5.711 | 0.000 |
|  | CultivationPTS | 0.353 | 1.041 | 20 | 0.340 | 0.738 |
|  | Stage.L | -0.241 | 1.074 | 40 | -0.224 | 0.824 |
|  | Stage.Q | 5.365 | 1.074 | 40 | 4.997 | 0.000 |
|  | CultivationHTF:Stage.L | 4.759 | 1.518 | 40 | 3.135 | 0.003 |
|  | CultivationPT:Stage.L | 2.336 | 1.518 | 40 | 1.539 | 0.132 |
|  | CultivationPTF:Stage.L | 3.947 | 1.518 | 40 | 2.600 | 0.013 |
|  | CultivationPTS:Stage.L | 2.738 | 1.518 | 40 | 1.804 | 0.079 |
|  | CultivationHTF:Stage.Q | -0.580 | 1.518 | 40 | -0.382 | 0.705 |
|  | CultivationPT:Stage.Q | -2.496 | 1.518 | 40 | -1.644 | 0.108 |
|  | CultivationPTF:Stage.Q | -2.458 | 1.518 | 40 | -1.619 | 0.113 |
|  | CultivationPTS:Stage.Q | -3.100 | 1.518 | 40 | -2.042 | 0.048 |

#### 2.1.3 Residual Diagnostic Plots


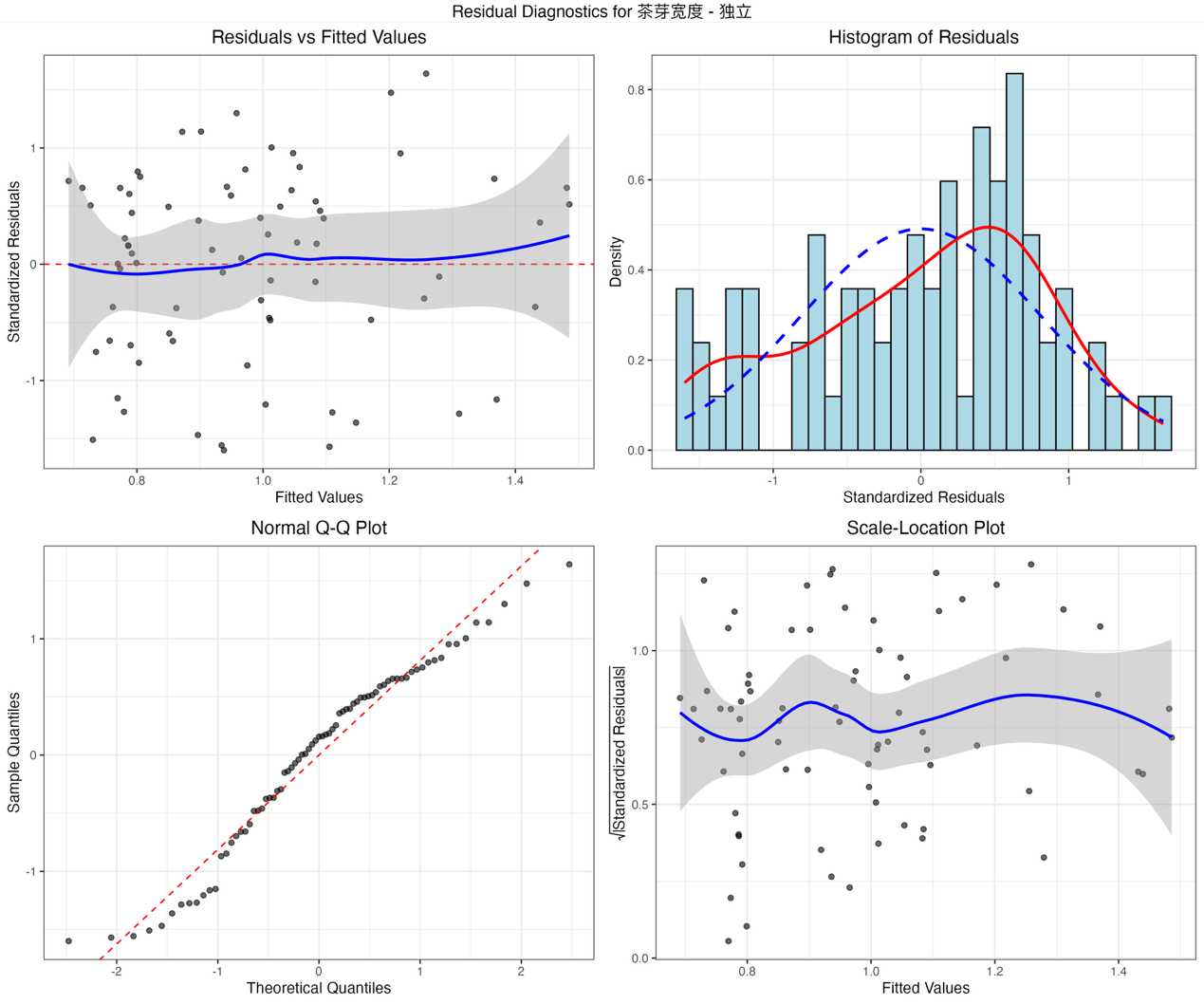


**Fig. S1.** Residual diagnostic plots for the best-fitted model of tea bud length (INDEP model). From left to right, top to bottom: residuals vs. fitted values, histogram of residuals with normal density curve, normal Q-Q plot, and scale-location plot.


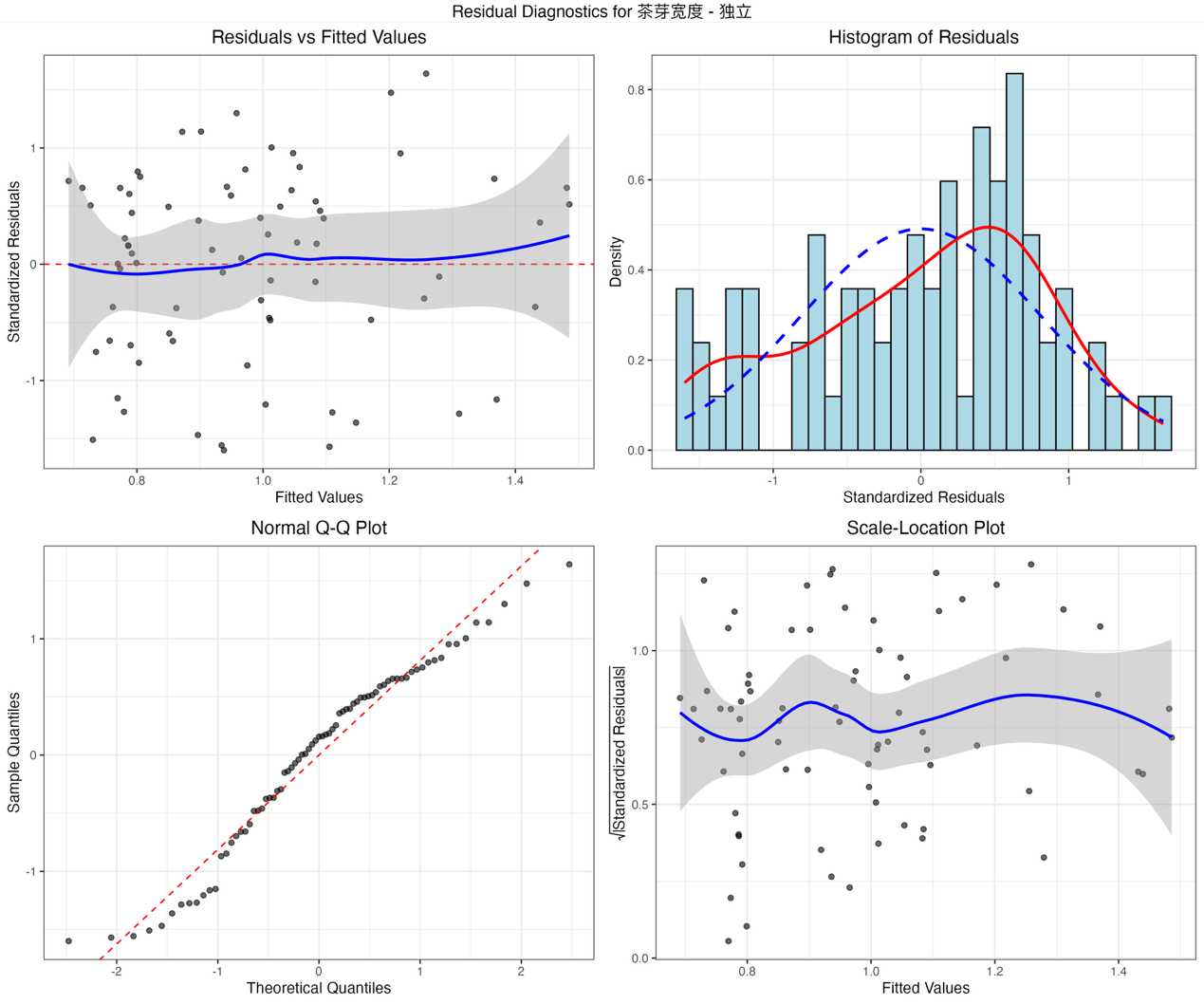


**Fig. S2.** Residual diagnostic plots for the best-fitted model of tea bud width (INDEP model). From left to right, top to bottom: residuals vs. fitted values, histogram of residuals with normal density curve, normal Q-Q plot, and scale-location plot.


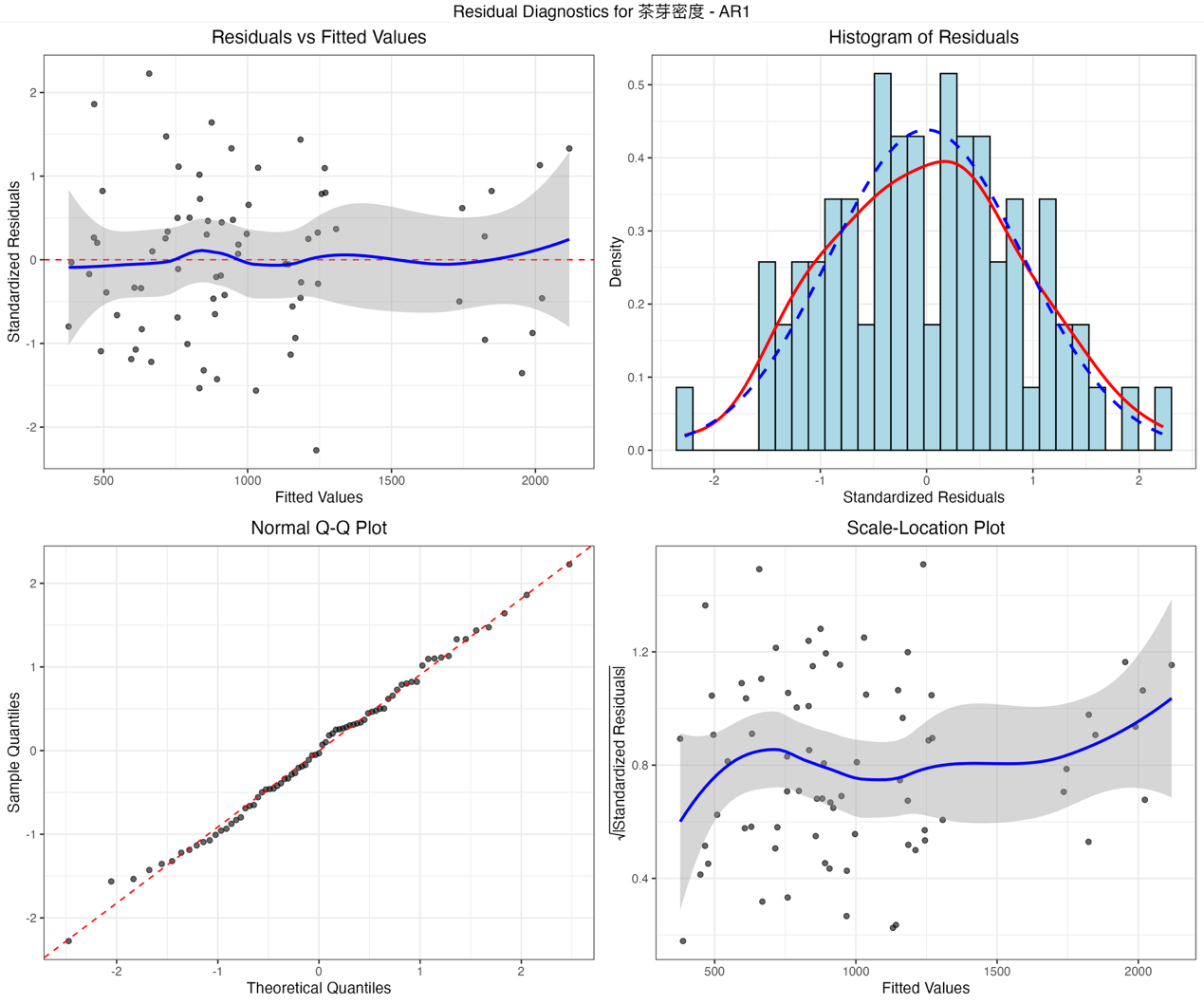


**Fig. S3.** Residual diagnostic plots for the best-fitted model of tea bud number (AR1 model). From left to right, top to bottom: residuals vs. fitted values, histogram of residuals with normal density curve, normal Q-Q plot, and scale-location plot.


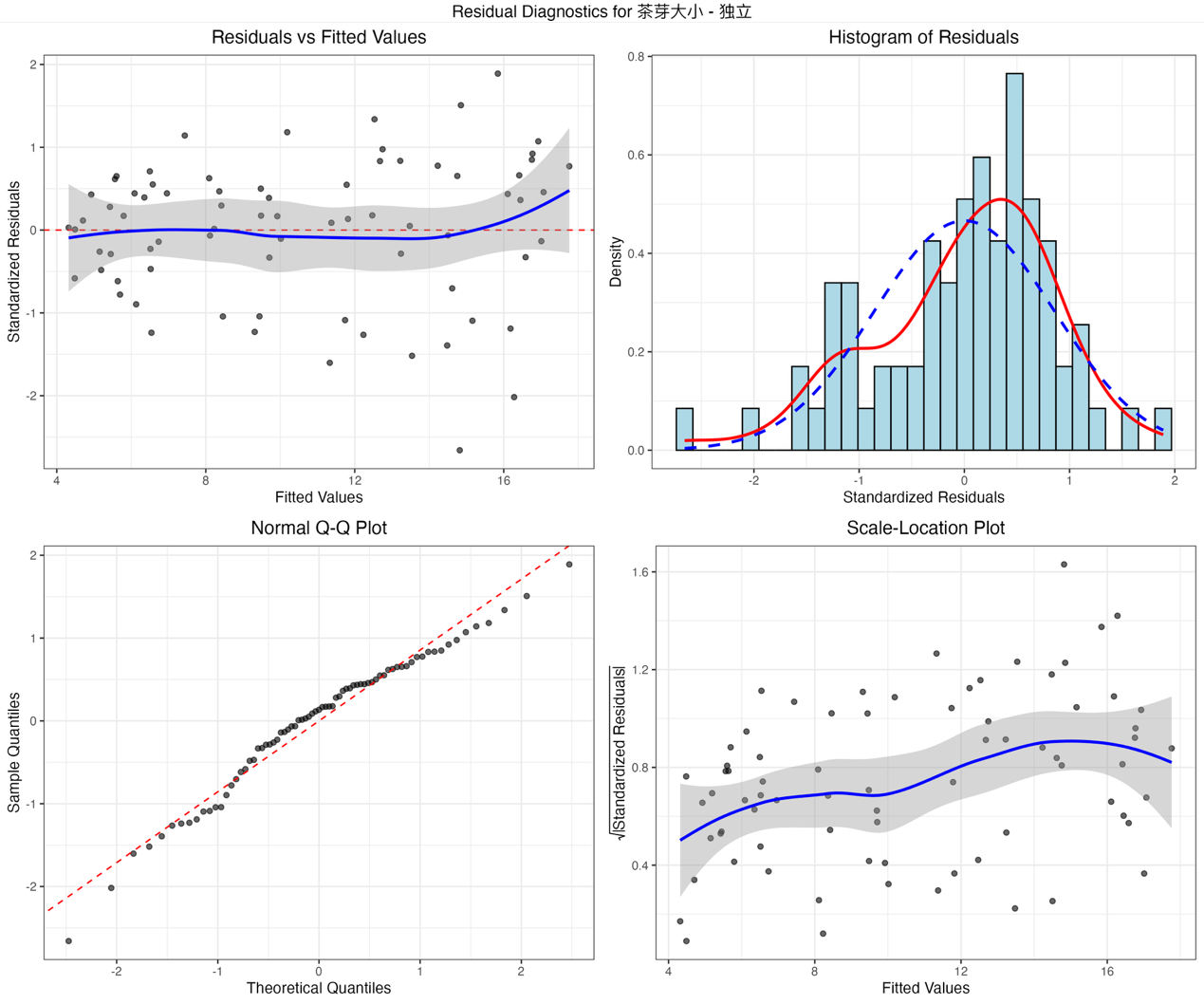


**Fig. S4.** Residual diagnostic plots for the best-fitted model of tea bud size (INDEP model). From left to right, top to bottom: residuals vs. fitted values, histogram of residuals with normal density curve, normal Q-Q plot, and scale-location plot.

#### 2.1.4 Paired t-tests for Growth Rates

**Table S5.** Paired t-test results comparing daily growth rates of tea bud morphological traits between early-to-mid (13 days) and mid-to-late (19 days) sprouting periods, overall and within each cultivation mode.

| **Metric** | **Cultivation_Mode** | **N_Pairs** | **Mean_Early** | **Mean_Late** | **Normality_W** | **Normality_p** | **Normality_Pass** | **t_value** | **df** | **p_value** | **Effect Size (Cohen's d)** | **Significance** |
| --- | --- | --- | --- | --- | --- | --- | --- | --- | --- | --- | --- | --- |
| Bud length | Overall | 25 | 0.108 | 0.484 | 0.954 | 0.304 | Yes | 10.808 | 24 | 0.000 | 2.162 | *** |
| Bud width | Overall | 25 | -0.019 | -0.002 | 0.963 | 0.473 | Yes | 5.065 | 24 | 0.000 | 1.013 | *** |
| Bud number | Overall | 25 | 29.274 | 23.710 | 0.941 | 0.156 | Yes | -0.812 | 24 | 0.425 | -0.162 | ns |
| Bud size | Overall | 25 | -0.206 | 0.328 | 0.953 | 0.300 | Yes | 7.595 | 24 | 0.000 | 1.519 | *** |
| Bud length | HT | 5 | 0.108 | 0.391 | 0.816 | 0.109 | Yes | 5.440 | 4 | 0.006 | 2.433 | ** |
| Bud width | HT | 5 | -0.033 | -0.002 | 0.972 | 0.887 | Yes | 4.592 | 4 | 0.010 | 2.054 | * |
| Bud number | HT | 5 | 5.855 | 18.480 | 0.954 | 0.769 | Yes | 0.967 | 4 | 0.388 | 0.433 | ns |
| Bud size | HT | 5 | -0.519 | 0.337 | 0.852 | 0.201 | Yes | 4.623 | 4 | 0.010 | 2.068 | ** |
| Bud length | HTF | 5 | 0.158 | 0.567 | 0.948 | 0.725 | Yes | 4.701 | 4 | 0.009 | 2.102 | ** |
| Bud width | HTF | 5 | -0.023 | 0.004 | 0.902 | 0.421 | Yes | 4.870 | 4 | 0.008 | 2.178 | ** |
| Bud number | HTF | 5 | 30.598 | 19.135 | 0.822 | 0.122 | Yes | -0.798 | 4 | 0.470 | -0.357 | ns |
| Bud size | HTF | 5 | -0.205 | 0.477 | 0.944 | 0.693 | Yes | 5.803 | 4 | 0.004 | 2.595 | ** |
| Bud length | PT | 5 | 0.095 | 0.545 | 0.941 | 0.674 | Yes | 4.833 | 4 | 0.008 | 2.161 | ** |
| Bud width | PT | 5 | -0.018 | -0.002 | 0.913 | 0.486 | Yes | 5.186 | 4 | 0.007 | 2.319 | ** |
| Bud number | PT | 5 | 28.547 | 51.930 | 0.852 | 0.200 | Yes | 1.237 | 4 | 0.284 | 0.553 | ns |
| Bud size | PT | 5 | -0.156 | 0.263 | 0.791 | 0.069 | Yes | 7.992 | 4 | 0.001 | 3.574 | ** |
| Bud length | PTF | 5 | 0.112 | 0.502 | 0.925 | 0.560 | Yes | 4.133 | 4 | 0.014 | 1.848 | * |
| Bud width | PTF | 5 | -0.011 | 0.000 | 0.941 | 0.676 | Yes | 2.331 | 4 | 0.080 | 1.042 | ns |
| Bud number | PTF | 5 | 59.829 | 30.526 | 0.856 | 0.213 | Yes | -2.578 | 4 | 0.061 | -1.153 | ns |
| Bud size | PTF | 5 | -0.072 | 0.325 | 0.966 | 0.847 | Yes | 3.641 | 4 | 0.022 | 1.628 | * |
| Bud length | PTS | 5 | 0.064 | 0.416 | 0.842 | 0.171 | Yes | 5.203 | 4 | 0.007 | 2.327 | ** |
| Bud width | PTS | 5 | -0.008 | -0.009 | 0.890 | 0.356 | Yes | -0.119 | 4 | 0.911 | -0.053 | ns |
| Bud number | PTS | 5 | 21.538 | -1.520 | 0.965 | 0.845 | Yes | -4.324 | 4 | 0.012 | -1.934 | * |
| Bud size | PTS | 5 | -0.077 | 0.239 | 0.943 | 0.689 | Yes | 1.702 | 4 | 0.164 | 0.761 | ns |


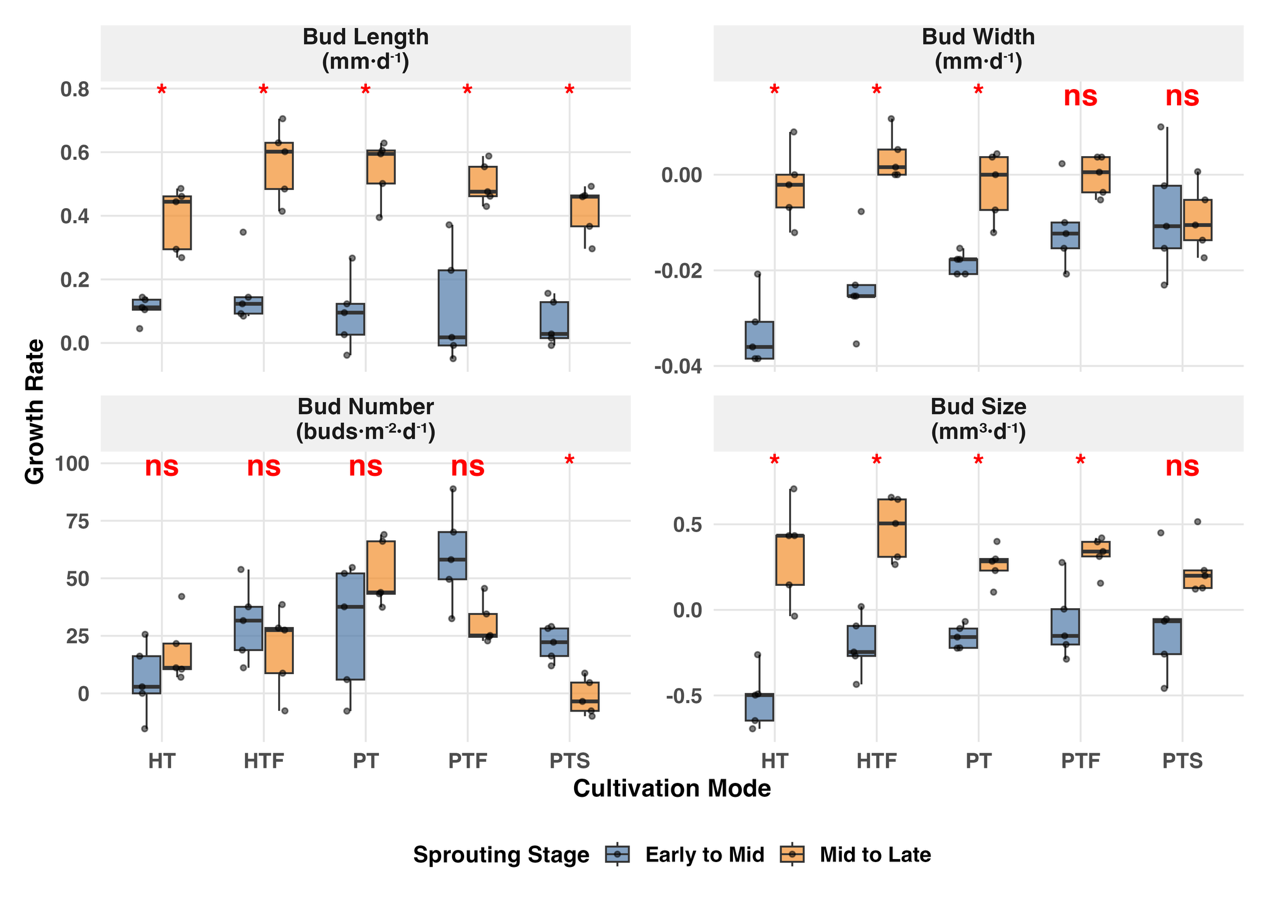


**Fig. S5.** Boxplots comparing daily growth rates of tea bud morphological traits between two successive sprouting periods within each cultivation mode (n = 5). Growth rates were calculated as daily absolute changes from early-to-mid (13 days) and mid-to-late (19 days) stages. Boxes represent interquartile range (IQR), horizontal lines denote medians, points indicate individual observations. Asterisks above each cultivation mode indicate significance levels from paired-sample t-tests (ns: not significant; **: P < 0.05*).

### 2.2 Part II: Effects of Cultivation Modes on Chemical Quality, Final Morphology, and Growth Rates

#### 2.2.1 Model Diagnostics for All Indicators

**Table S6.** Model assumption tests for mixed-effects models of individual and composite indicators. Tests include Shapiro-Wilk normality test, Levene’s homogeneity of variance test, and Durbin-Watson autocorrelation test.

|  |  | Shapiro_Wilk_W | Shapiro_Wilk_p | Normality_Interpretation | Levene_F | Levene_p | Homogeneity_Interpretation | Durbin_Watson_DW | Durbin_Watson_p | Autocorrelation_Interpretation |
| --- | --- | --- | --- | --- | --- | --- | --- | --- | --- | --- |
| Individual indicator | Free amino acids | 0.935 | 0.114 | Normally | 0.833 | 0.520 | Homogeneous | 2.198 | 0.748 | No |
|  | Tea polyphenols | 0.894 | 0.013 | NOT normally | 2.040 | 0.127 | Homogeneous | 1.904 | 0.288 | No |
|  | Phenol/amino acid ratio | 0.965 | 0.515 | Normally | 1.772 | 0.174 | Homogeneous | 2.373 | 0.898 | No |
|  | Bud length | 0.934 | 0.109 | Normally | 0.543 | 0.706 | Homogeneous | 1.582 | 0.044 | Autocorrelation |
|  | Bud width | 0.887 | 0.010 | NOT normally | 0.395 | 0.810 | Homogeneous | 2.184 | 0.696 | No |
|  | Bud number | 0.977 | 0.826 | Normally | 0.819 | 0.528 | Homogeneous | 1.851 | 0.200 | No |
|  | Bud size | 0.928 | 0.077 | Normally | 0.404 | 0.803 | Homogeneous | 2.092 | 0.512 | No |
|  | Length growth | 0.948 | 0.226 | Normally | 2.211 | 0.104 | Homogeneous | 2.358 | 0.902 | No |
|  | Width growth | 0.964 | 0.510 | Normally | 0.315 | 0.864 | Homogeneous | 2.364 | 0.890 | No |
|  | Number growth | 0.931 | 0.091 | Normally | 1.310 | 0.300 | Homogeneous | 2.618 | 0.426 | No |
|  | Size growth | 0.948 | 0.224 | Normally | 0.057 | 0.993 | Homogeneous | 2.668 | 0.354 | No |
|  |  |  |  |  |  |  |  |  |  |  |
| Composite indicator | Chemical_PC1 | 0.909 | 0.029 | NOT normally | 2.382 | 0.086 | Homogeneous | 2.229 | 0.802 | No |
|  | Morphological_PC1 | 0.956 | 0.345 | Normally | 0.327 | 0.857 | Homogeneous | 2.120 | 0.558 | No |
|  | Growth_PC1 | 0.955 | 0.321 | Normally | 0.109 | 0.978 | Homogeneous | 2.809 | 0.176 | No |

**Table S7.** Type III ANOVA results for mixed-effects models showing the effects of cultivation mode on individual chemical, morphological, and growth rate indicators, as well as composite indicators derived from principal component analysis (PCA).

|  | Indicator | Factor | NumDF | DenDF | F value | Pr(>F) |
| --- | --- | --- | --- | --- | --- | --- |
| Individual indicator | Tea polyphenols | Cultivation | 4 | 20 | 4.513 | 0.009 |
|  | Free amino acids | Cultivation | 4 | 20 | 8.153 | 0.000 |
|  | Phenol/amino acid ratio | Cultivation | 4 | 20 | 7.453 | 0.001 |
|  | Bud length | Cultivation | 4 | 20 | 5.118 | 0.005 |
|  | Bud width | Cultivation | 4 | 16 | 9.557 | 0.000 |
|  | Bud number | Cultivation | 4 | 20 | 40.621 | 0.000 |
|  | Bud size | Cultivation | 4 | 16 | 6.048 | 0.004 |
|  | Length growth | Cultivation | 4 | 16 | 6.254 | 0.003 |
|  | Width growth | Cultivation | 4 | 16 | 8.894 | 0.001 |
|  | Number growth | Cultivation | 4 | 20 | 31.743 | 0.000 |
|  | Size growth | Cultivation | 4 | 16 | 4.548 | 0.012 |
|  |  |  |  |  |  |  |
| Composite indicator | Chemical_PC1 | Cultivation | 4 | 20 | 8.061 | 0.000 |
|  | Morphological_PC1 | Cultivation | 4 | 16 | 17.014 | 0.000 |
|  | Growth_PC1 | Cultivation | 4 | 16 | 13.186 | 0.000 |

**Table S8.** Parameter coefficients for mixed-effects models of individual and composite indicators, including estimates, standard errors, degrees of freedom, t-values, and p-values.

|  |  |  | Estimate | Std. Error | df | t value | Pr(>\|t\|) |
| --- | --- | --- | --- | --- | --- | --- | --- |
| Individual indicator | Tea polyphenols | (Intercept) | 18.594 | 0.460 | 20 | 40.385 | 0.000 |
|  |  | CultivationHTF | -1.092 | 0.651 | 20 | -1.678 | 0.109 |
|  |  | CultivationPT | -1.899 | 0.651 | 20 | -2.916 | 0.009 |
|  |  | CultivationPTF | -0.795 | 0.651 | 20 | -1.221 | 0.236 |
|  |  | CultivationPTS | -2.520 | 0.651 | 20 | -3.870 | 0.001 |
|  | Free amino acids | (Intercept) | 2.546 | 0.284 | 20 | 8.966 | 0.000 |
|  |  | CultivationHTF | -0.100 | 0.402 | 20 | -0.249 | 0.806 |
|  |  | CultivationPT | 1.144 | 0.402 | 20 | 2.849 | 0.010 |
|  |  | CultivationPTF | 0.502 | 0.402 | 20 | 1.249 | 0.226 |
|  |  | CultivationPTS | 1.825 | 0.402 | 20 | 4.545 | 0.000 |
|  | Phenol/aminoacid ratio | (Intercept) | 7.411 | 0.604 | 20 | 12.264 | 0.000 |
|  |  | CultivationHTF | -0.029 | 0.855 | 20 | -0.033 | 0.974 |
|  |  | CultivationPT | -2.749 | 0.855 | 20 | -3.217 | 0.004 |
|  |  | CultivationPTF | -1.051 | 0.855 | 20 | -1.230 | 0.233 |
|  |  | CultivationPTS | -3.670 | 0.855 | 20 | -4.294 | 0.000 |
|  | Bud length | (Intercept) | 16.606 | 0.547 | 20 | 30.343 | 0.000 |
|  |  | CultivationHTF | 3.104 | 0.774 | 20 | 4.011 | 0.001 |
|  |  | CultivationPT | 1.491 | 0.774 | 20 | 1.926 | 0.068 |
|  |  | CultivationPTF | 1.248 | 0.774 | 20 | 1.613 | 0.122 |
|  |  | CultivationPTS | 0.210 | 0.774 | 20 | 0.272 | 0.788 |
|  | Bud width | (Intercept) | 0.968 | 0.043 | 16 | 22.582 | 0.000 |
|  |  | CultivationHTF | -0.110 | 0.052 | 16 | -2.095 | 0.052 |
|  |  | CultivationPT | -0.243 | 0.052 | 16 | -4.656 | 0.000 |
|  |  | CultivationPTF | -0.160 | 0.052 | 16 | -3.061 | 0.007 |
|  |  | CultivationPTS | 0.034 | 0.052 | 16 | 0.659 | 0.519 |
|  | Bud number | (Intercept) | 1168.889 | 72.883 | 20 | 16.038 | 0.000 |
|  |  | CultivationHTF | 88.000 | 103.072 | 20 | 0.854 | 0.403 |
|  |  | CultivationPT | 851.111 | 103.072 | 20 | 8.257 | 0.000 |
|  |  | CultivationPTF | 626.667 | 103.072 | 20 | 6.080 | 0.000 |
|  |  | CultivationPTS | -271.111 | 103.072 | 20 | -2.630 | 0.016 |
|  | Bud size | (Intercept) | 15.954 | 1.414 | 17 | 11.287 | 0.000 |
|  |  | CultivationHTF | -1.313 | 1.756 | 16 | -0.748 | 0.465 |
|  |  | CultivationPT | -6.376 | 1.756 | 16 | -3.631 | 0.002 |
|  |  | CultivationPTF | -4.158 | 1.756 | 16 | -2.368 | 0.031 |
|  |  | CultivationPTS | 1.024 | 1.756 | 16 | 0.583 | 0.568 |
|  | Length growth | (Intercept) | 0.276 | 0.023 | 19 | 11.887 | 0.000 |
|  |  | CultivationHTF | 0.125 | 0.031 | 16 | 3.968 | 0.001 |
|  |  | CultivationPT | 0.086 | 0.031 | 16 | 2.730 | 0.015 |
|  |  | CultivationPTF | 0.067 | 0.031 | 16 | 2.142 | 0.048 |
|  |  | CultivationPTS | -0.003 | 0.031 | 16 | -0.097 | 0.924 |
|  | Width growth | (Intercept) | -0.015 | 0.002 | 10 | -8.303 | 0.000 |
|  |  | CultivationHTF | 0.008 | 0.002 | 16 | 4.258 | 0.001 |
|  |  | CultivationPT | 0.006 | 0.002 | 16 | 3.384 | 0.004 |
|  |  | CultivationPTF | 0.010 | 0.002 | 16 | 5.746 | 0.000 |
|  |  | CultivationPTS | 0.006 | 0.002 | 16 | 3.373 | 0.004 |
|  | Number growth | (Intercept) | 13.351 | 2.854 | 20 | 4.678 | 0.000 |
|  |  | CultivationHTF | 10.441 | 4.036 | 20 | 2.587 | 0.018 |
|  |  | CultivationPT | 29.080 | 4.036 | 20 | 7.205 | 0.000 |
|  |  | CultivationPTF | 29.080 | 4.036 | 20 | 7.205 | 0.000 |
|  |  | CultivationPTS | -5.503 | 4.036 | 20 | -1.363 | 0.188 |
|  | Size growth | (Intercept) | -0.011 | 0.054 | 10 | -0.196 | 0.848 |
|  |  | CultivationHTF | 0.210 | 0.053 | 16 | 3.950 | 0.001 |
|  |  | CultivationPT | 0.103 | 0.053 | 16 | 1.939 | 0.070 |
|  |  | CultivationPTF | 0.174 | 0.053 | 16 | 3.276 | 0.005 |
|  |  | CultivationPTS | 0.121 | 0.053 | 16 | 2.273 | 0.037 |
|  |  | | | | | | |
| Composite indicator | Chemical_PC1 | (Intercept) | 1.439 | 0.501 | 20 | 2.871 | 0.009 |
|  |  | CultivationHTF | -0.405 | 0.709 | 20 | -0.572 | 0.574 |
|  |  | CultivationPT | -2.403 | 0.709 | 20 | -3.391 | 0.003 |
|  |  | CultivationPTF | -0.990 | 0.709 | 20 | -1.396 | 0.178 |
|  |  | CultivationPTS | -3.395 | 0.709 | 20 | -4.791 | 0.000 |
|  | Morphological_PC1 | (Intercept) | 1.145 | 0.432 | 16.344 | 2.651 | 0.017 |
|  |  | CultivationHTF | -0.989 | 0.534 | 16 | -1.854 | 0.082 |
|  |  | CultivationPT | -3.178 | 0.534 | 16 | -5.955 | 0.000 |
|  |  | CultivationPTF | -2.185 | 0.534 | 16 | -4.094 | 0.001 |
|  |  | CultivationPTS | 0.627 | 0.534 | 16 | 1.176 | 0.257 |
|  | Growth_PC1 | (Intercept) | -1.889 | 0.524 | 9.550 | -3.604 | 0.005 |
|  |  | CultivationHTF | 2.927 | 0.512 | 16 | 5.719 | 0.000 |
|  |  | CultivationPT | 2.393 | 0.512 | 16 | 4.675 | 0.000 |
|  |  | CultivationPTF | 3.048 | 0.512 | 16 | 5.956 | 0.000 |
|  |  | CultivationPTS | 1.074 | 0.512 | 16 | 2.098 | 0.052 |

#### 2.2.2 Residual Diagnostic Plots for Individual Indicators


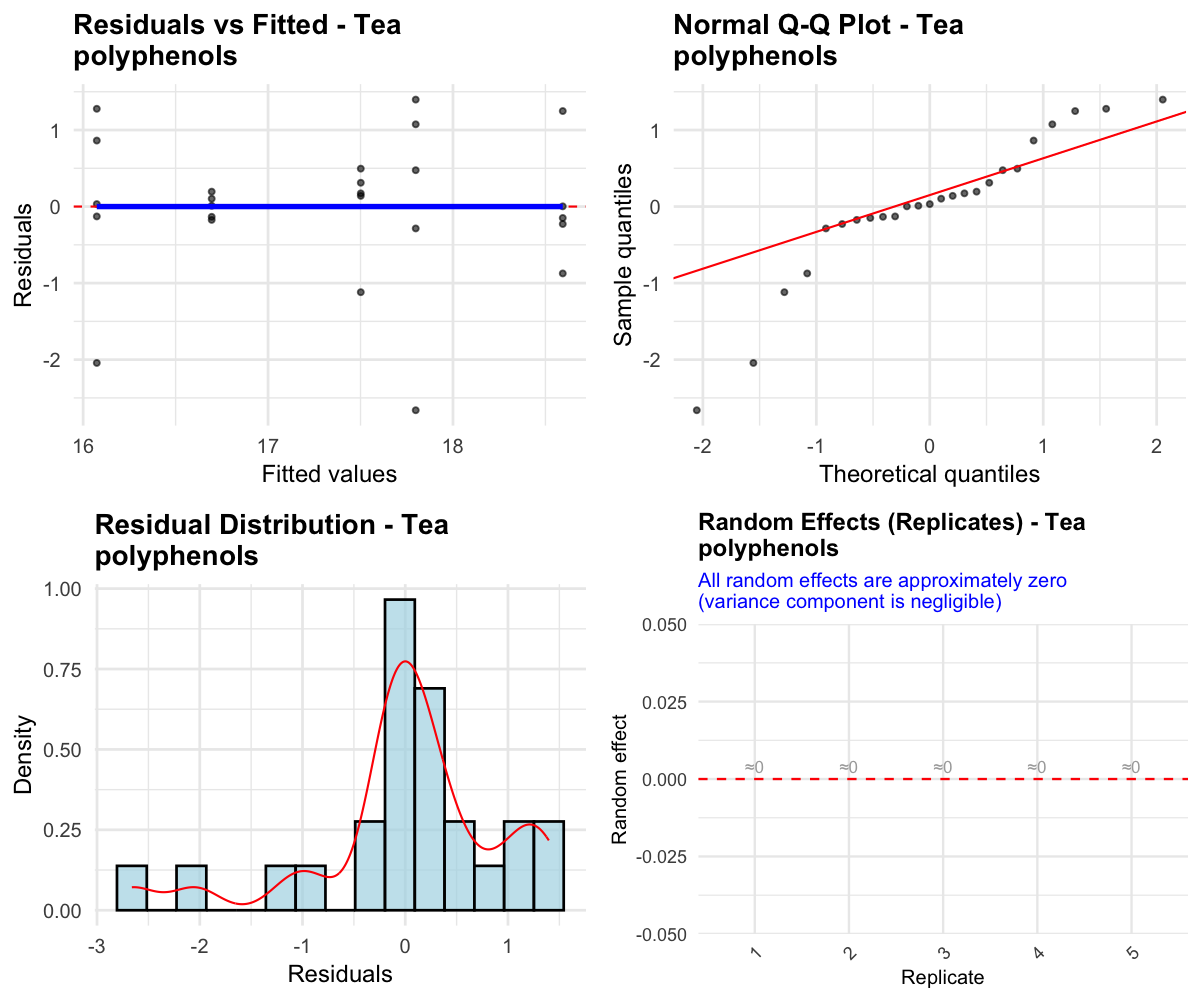


**Fig. S6.** Residual diagnostic plots for the mixed-effects model of tea polyphenol content.


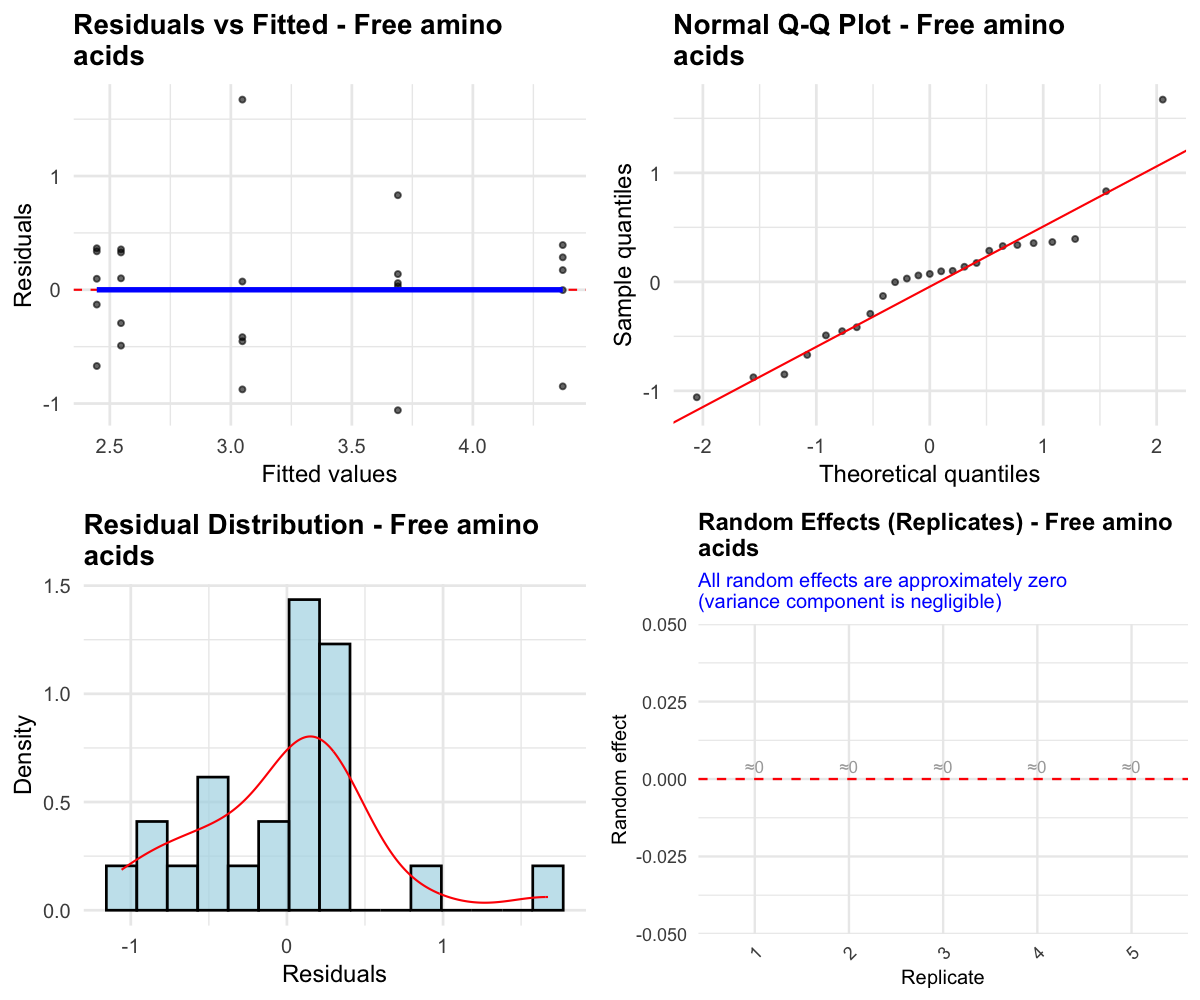


**Fig. S7.** Residual diagnostic plots for the mixed-effects model of free amino acid content.


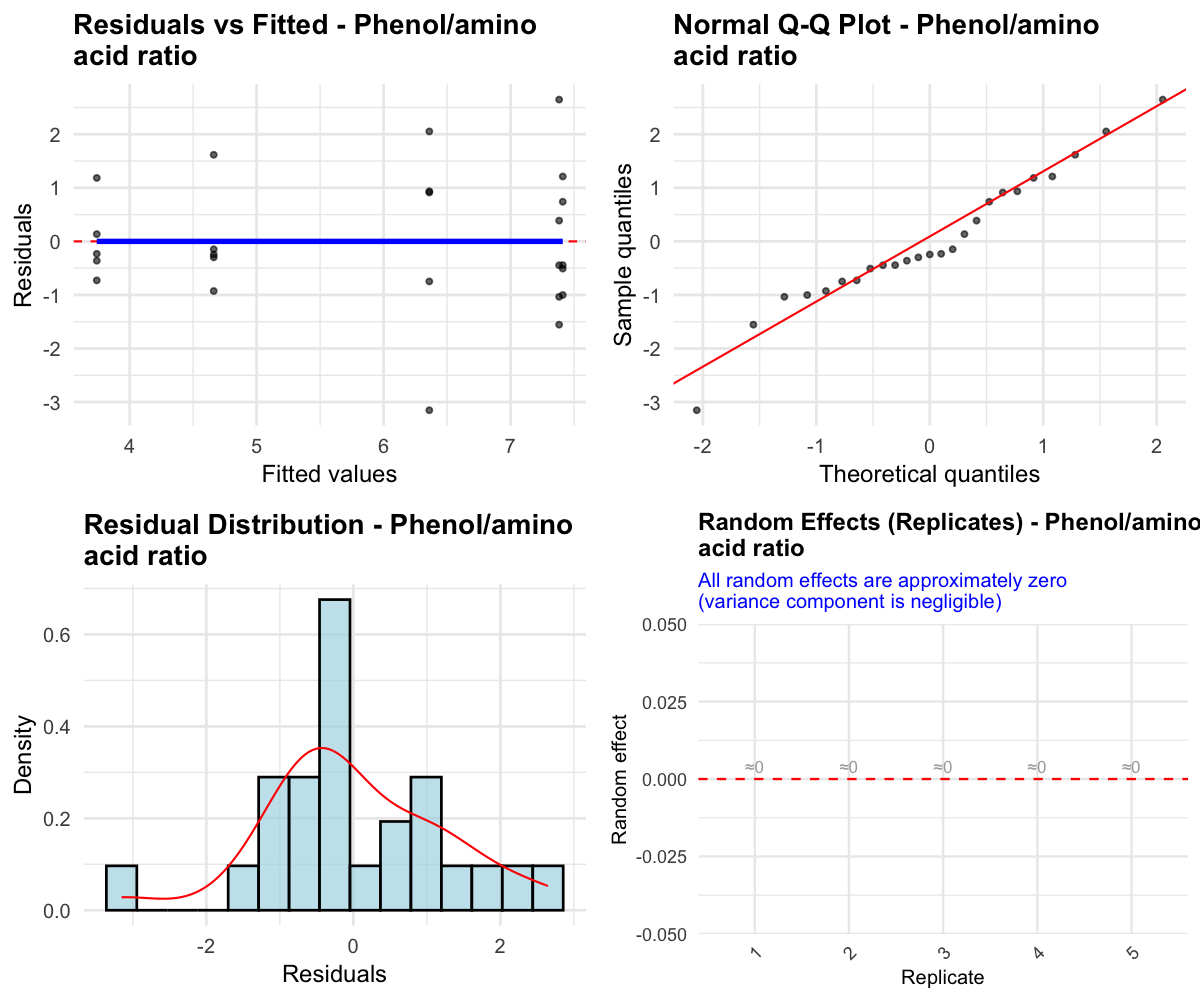


**Fig. S8.** Residual diagnostic plots for the mixed-effects model of phenol/amino acid ratio.


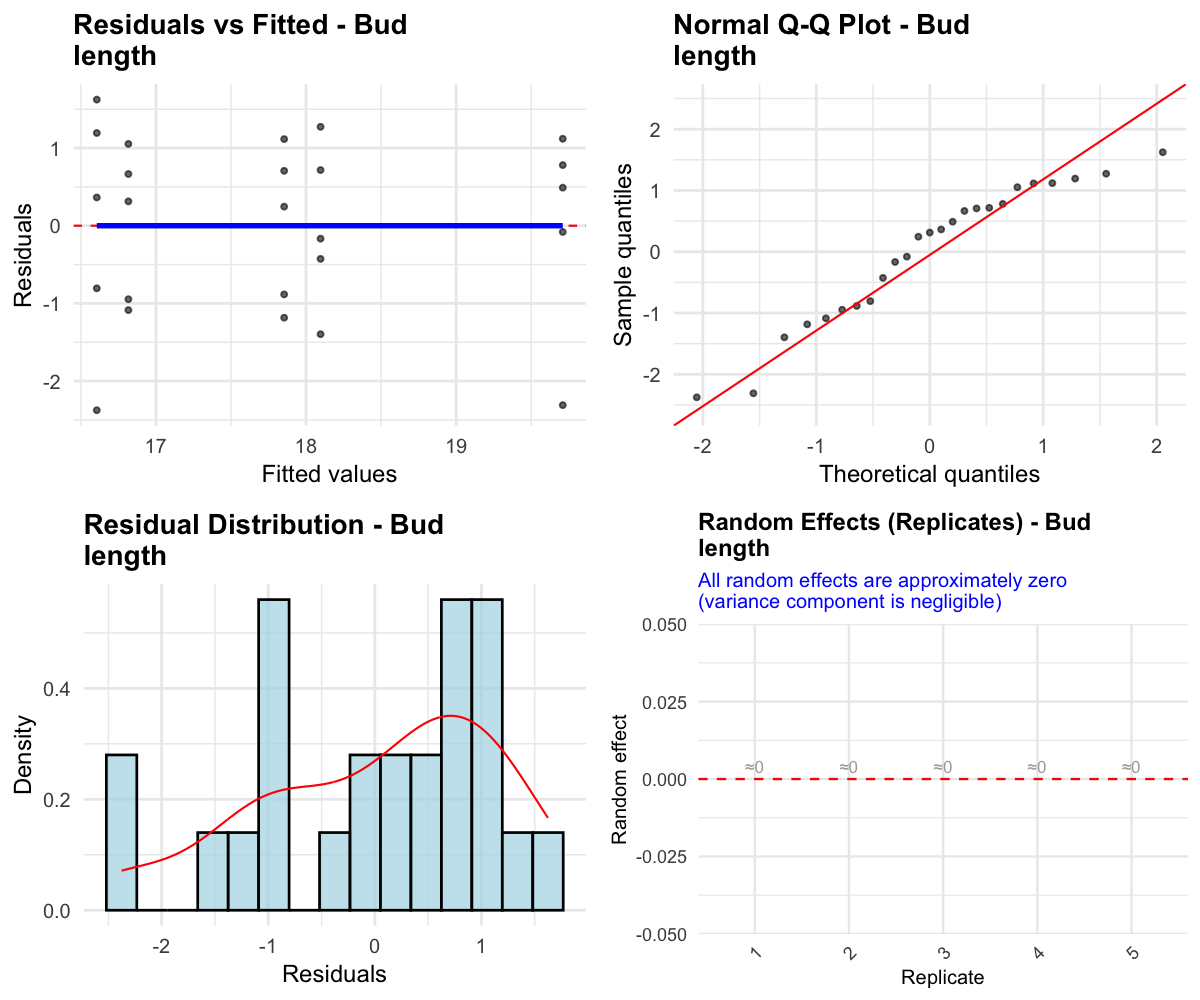


**Fig. S9.** Residual diagnostic plots for the mixed-effects model of final tea bud length.


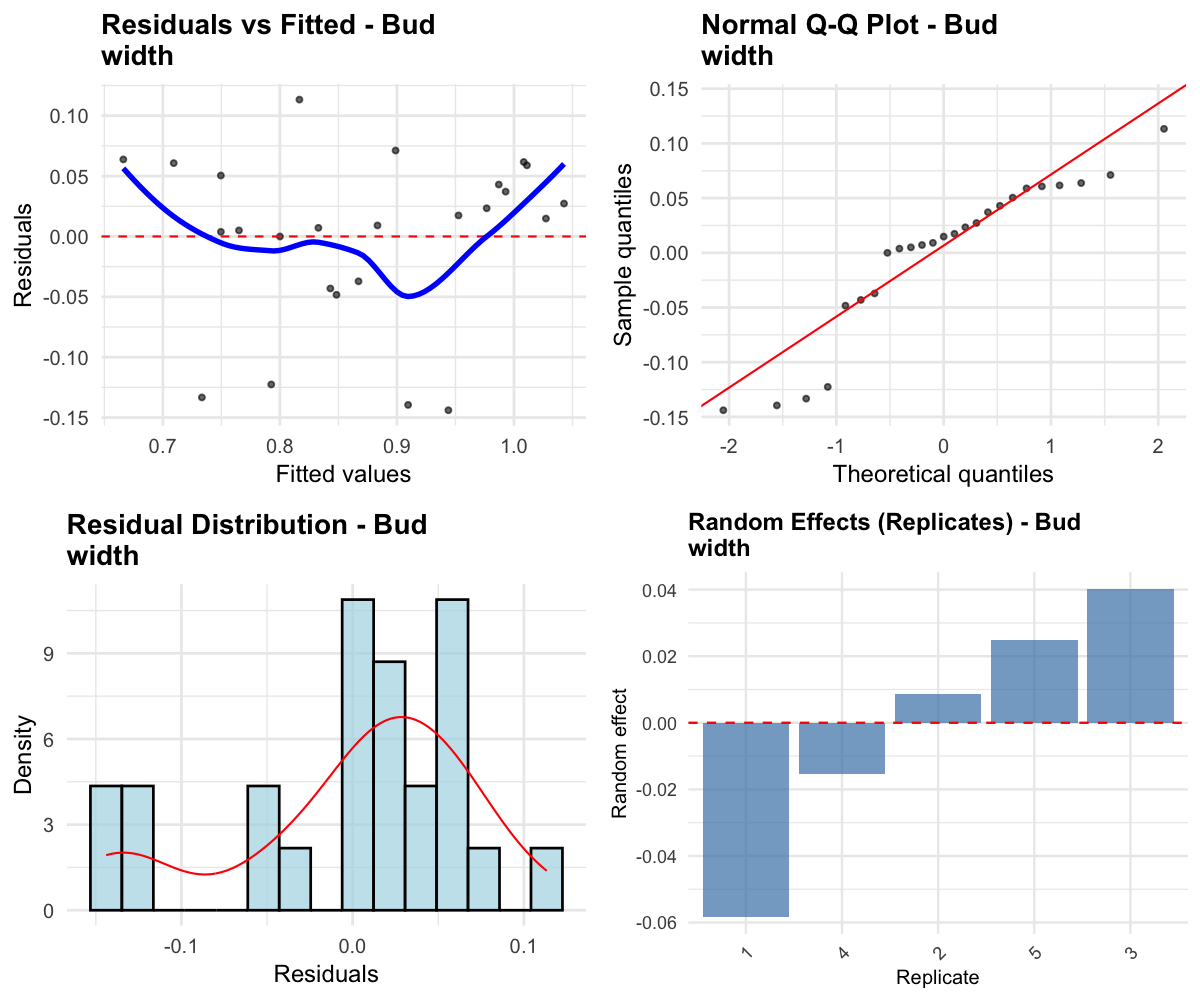


**Fig. S10.** Residual diagnostic plots for the mixed-effects model of final tea bud width.


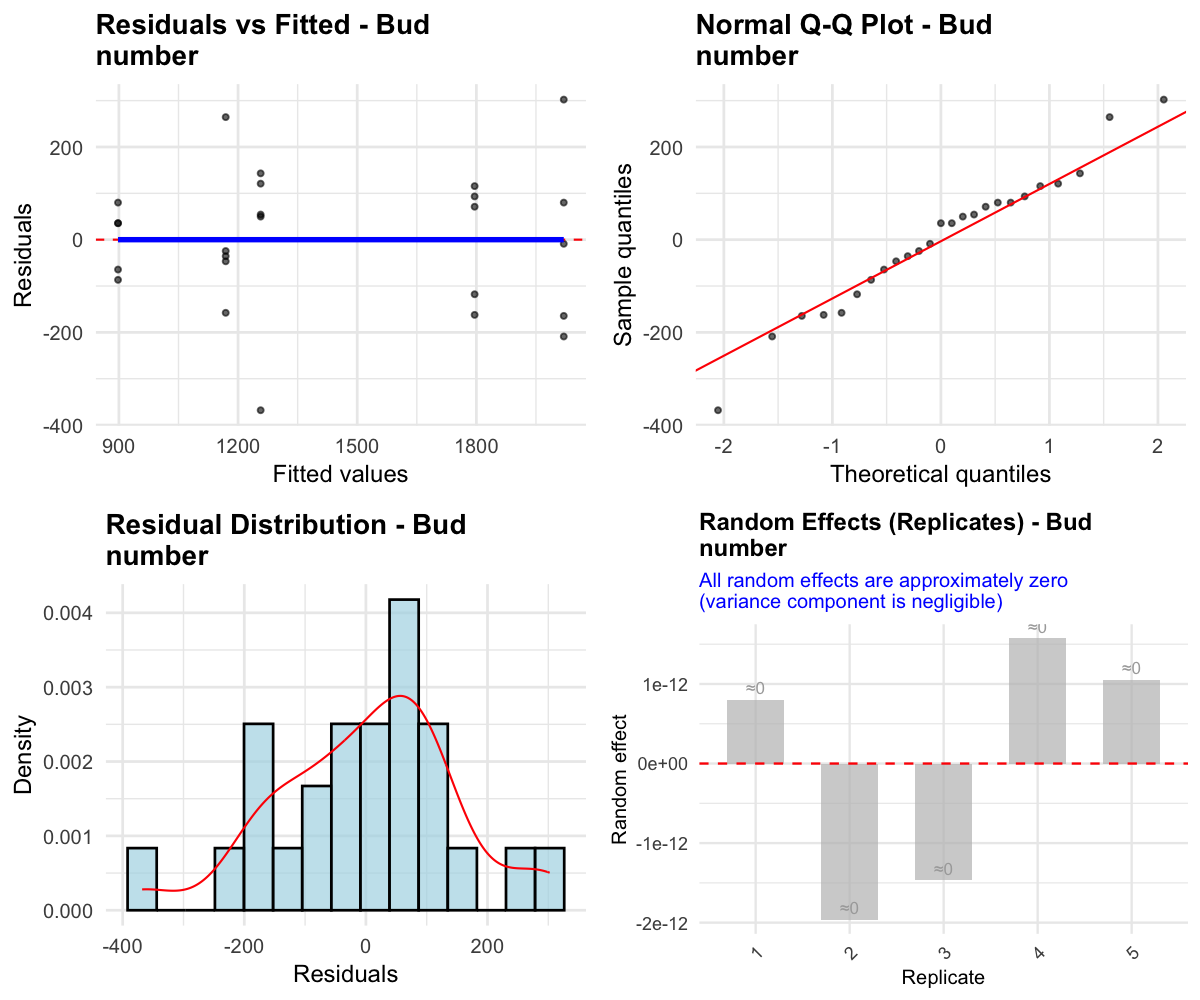


**Fig. S11.** Residual diagnostic plots for the mixed-effects model of final tea bud number.


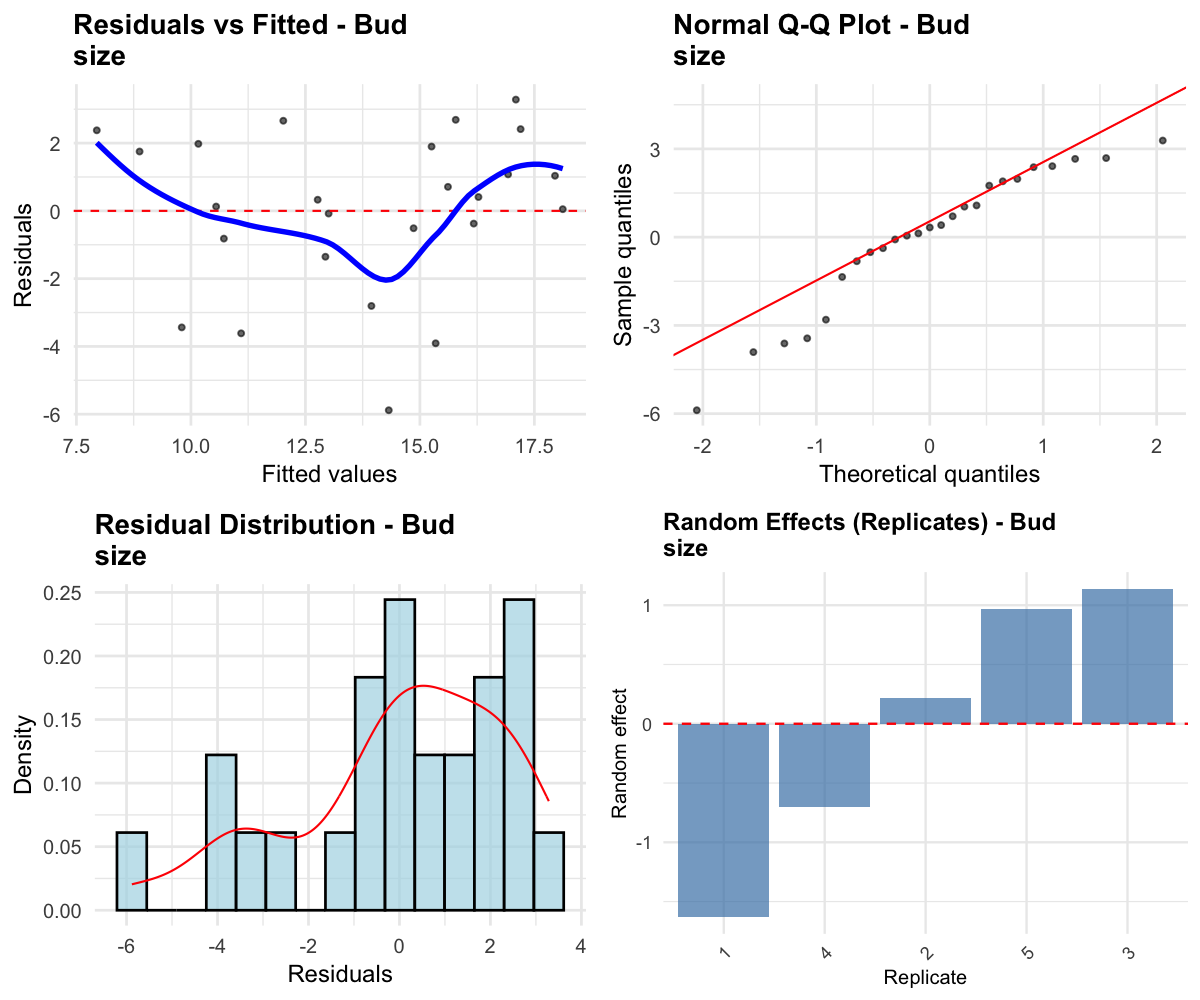


**Fig. S12.** Residual diagnostic plots for the mixed-effects model of final tea bud size.


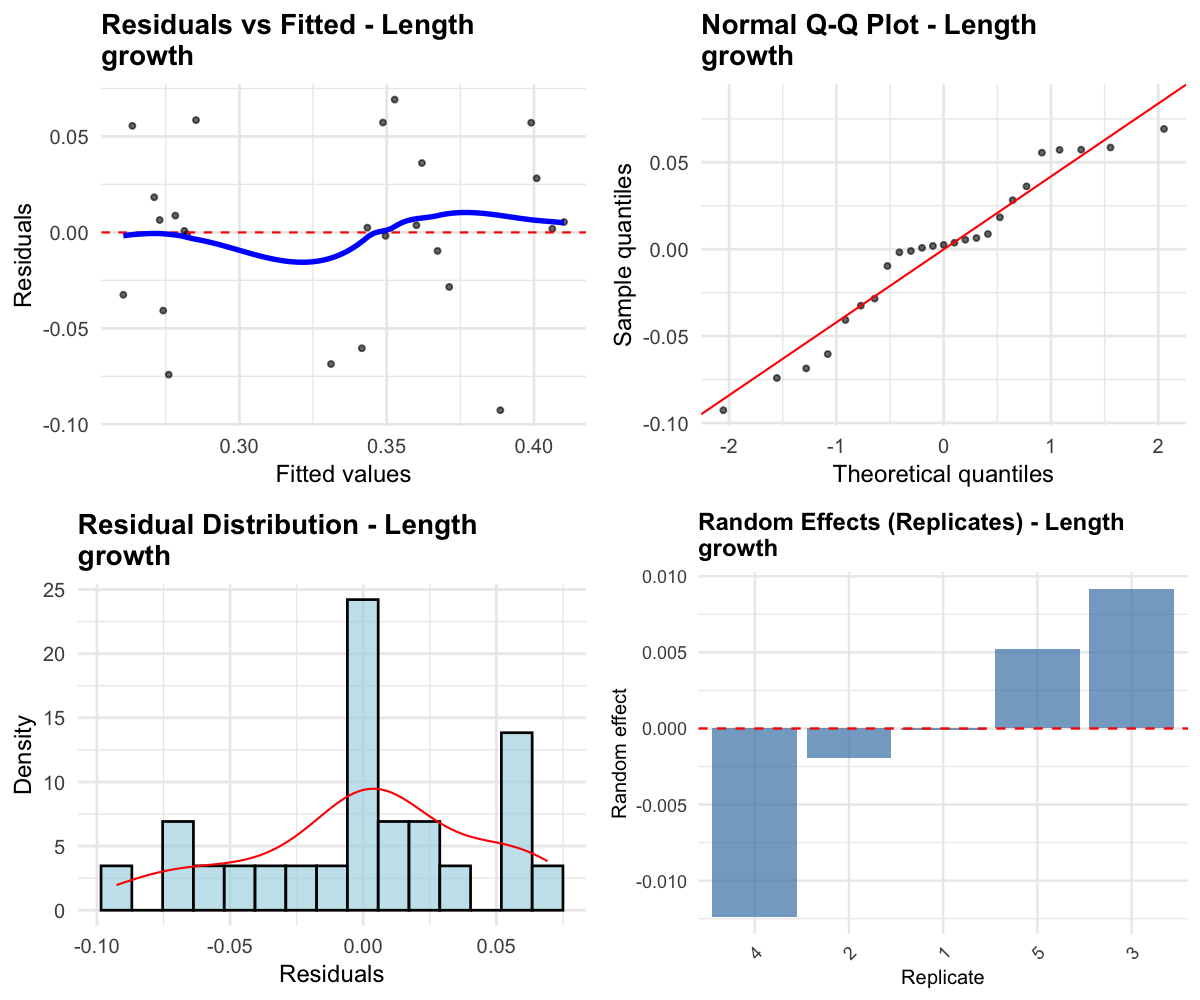


**Fig. S13.** Residual diagnostic plots for the mixed-effects model of length growth rate.


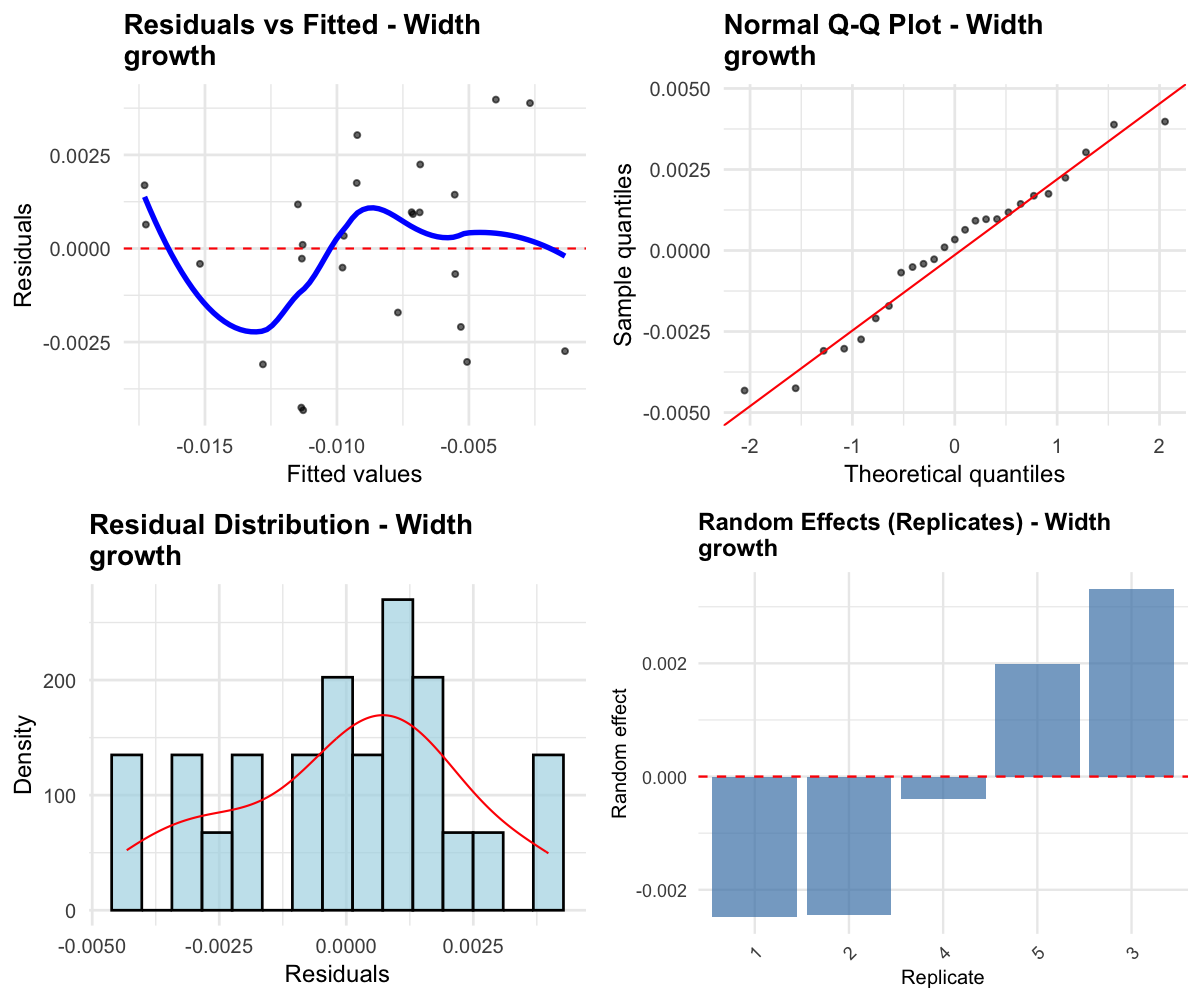


**Fig. S14.** Residual diagnostic plots for the mixed-effects model of width growth rate.


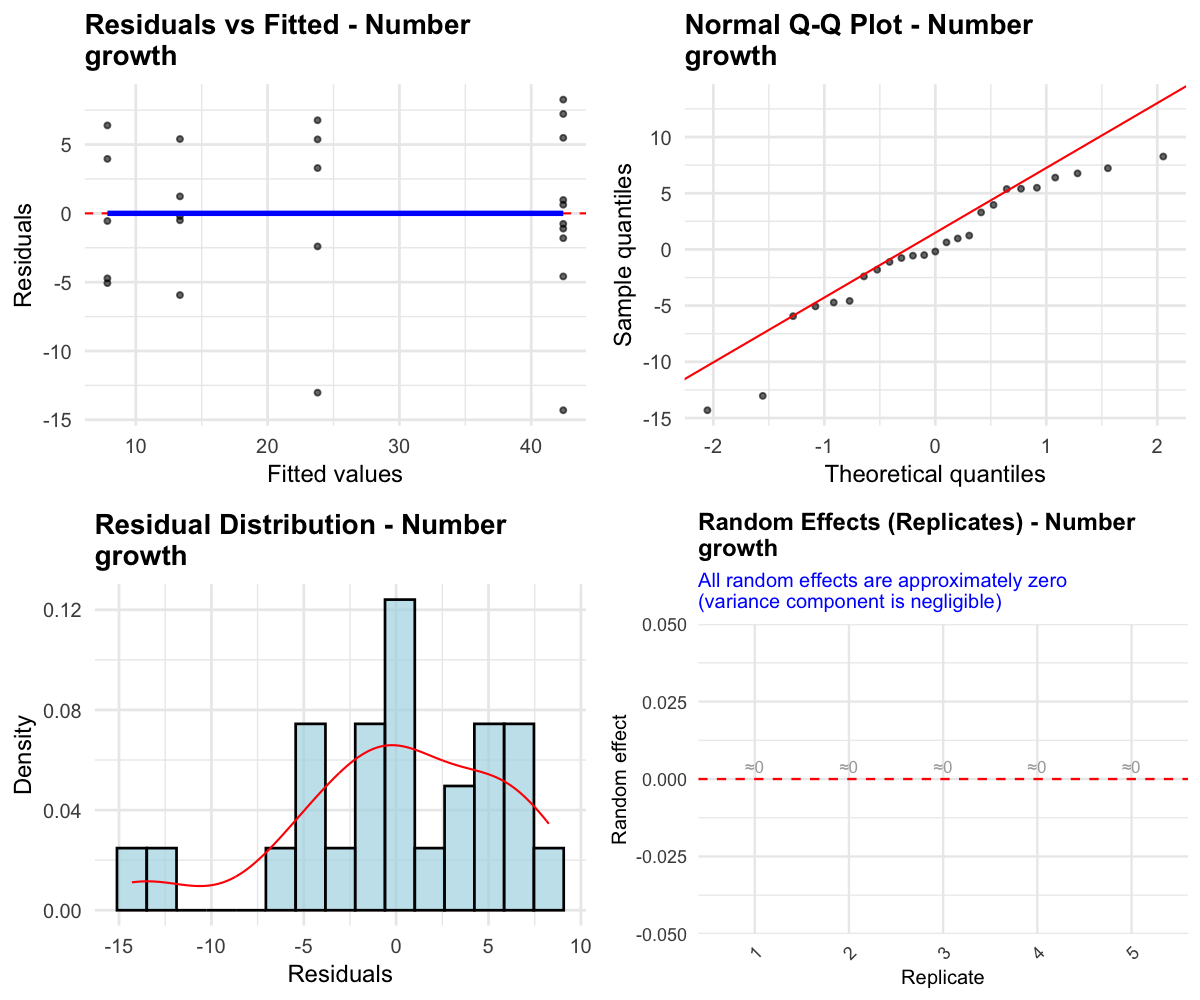


**Fig. S15.** Residual diagnostic plots for the mixed-effects model of number growth rate.


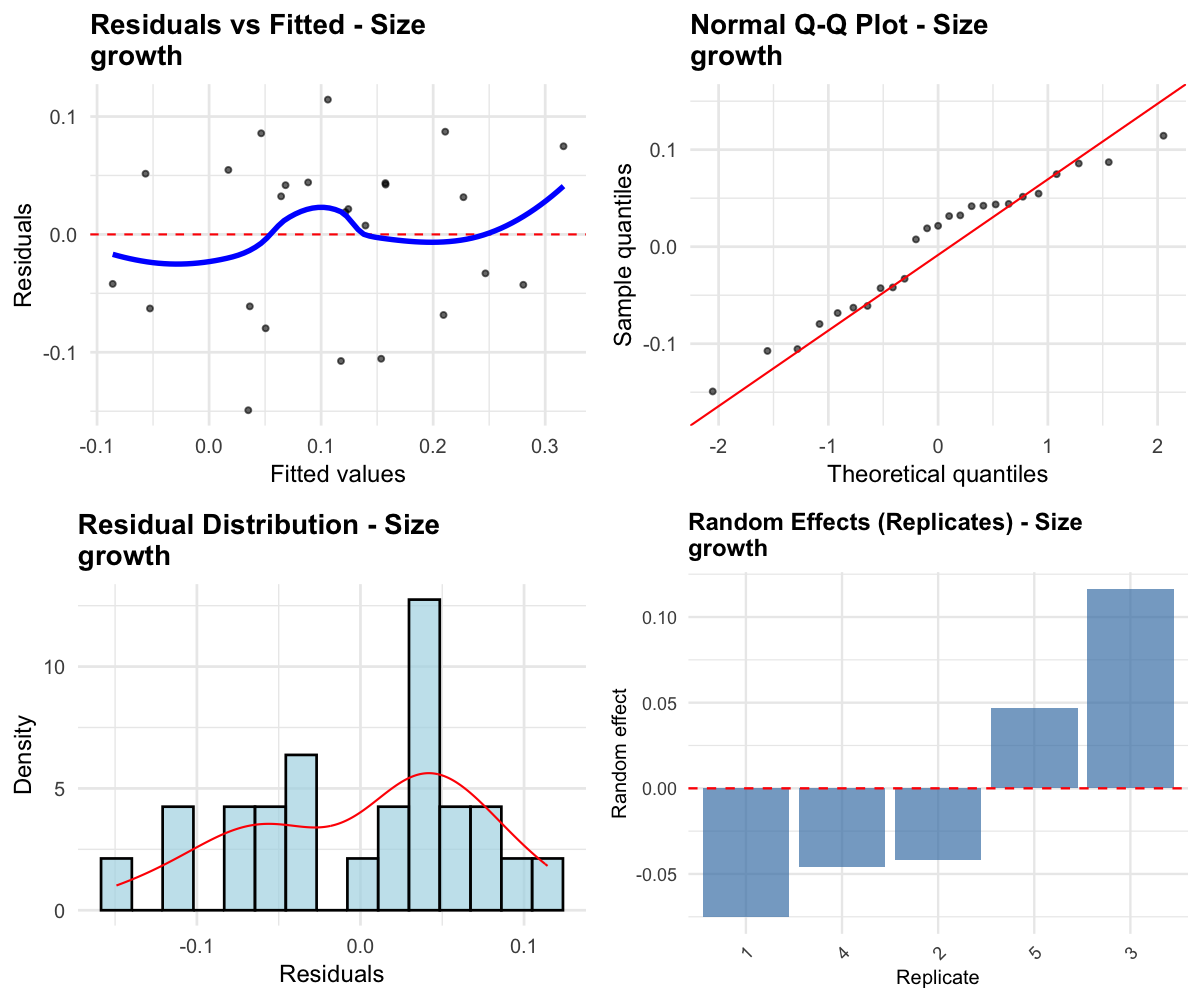


**Fig. S16.** Residual diagnostic plots for the mixed-effects model of size growth rate.

#### 2.2.3 Principal Component Analysis Results

**Table S9.** Eigenvalues and variance explained by the first three principal components for three indicator groups: chemical indicators (3 variables), morphological indicators (4 variables), and growth rate indicators (4 variables).

| Indicator groups | Principal component | Eigenvalue | Percentage of variance (%) | Cumulative percentage of variance (%) |
| --- | --- | --- | --- | --- |
| Chemical | PC1 | 2.62 | 87.46 | 87.46 |
|  | PC2 | 0.35 | 11.55 | 99.00 |
|  | PC3 | 0.03 | 1.00 | 100.00 |
|  |  |  |  |  |
| Morphological | PC1 | 2.68 | 67.11 | 67.11 |
|  | PC2 | 1.02 | 25.57 | 92.68 |
|  | PC3 | 0.29 | 7.23 | 99.91 |
|  |  |  |  |  |
| Growth | PC1 | 2.48 | 62.01 | 62.01 |
|  | PC2 | 0.93 | 23.37 | 85.37 |
|  | PC3 | 0.55 | 13.64 | 99.01 |

**Table S10.** Contributions of each original variable to the first principal component (PC1) for three indicator groups, expressed as percentages.

| Indicator groups | Indicator | Percentage of variance for PC1 (%) |
| --- | --- | --- |
| Chemical | Free amino acids | 35.43 |
|  | Tea polyphenols | 28.71 |
|  | Phenol/amino acid ratio | 35.86 |
|  |  |  |
| Morphological | Bud length | 0.98 |
|  | Bud width | 35.39 |
|  | Bud number | 29.68 |
|  | Bud size | 33.95 |
|  |  |  |
| Growth | Length growth | 24.83 |
|  | Width growth | 31.05 |
|  | Number growth | 11.08 |
|  | Size growth | 33.03 |

#### 2.2.4 Residual Diagnostic Plots for Composite Indicators


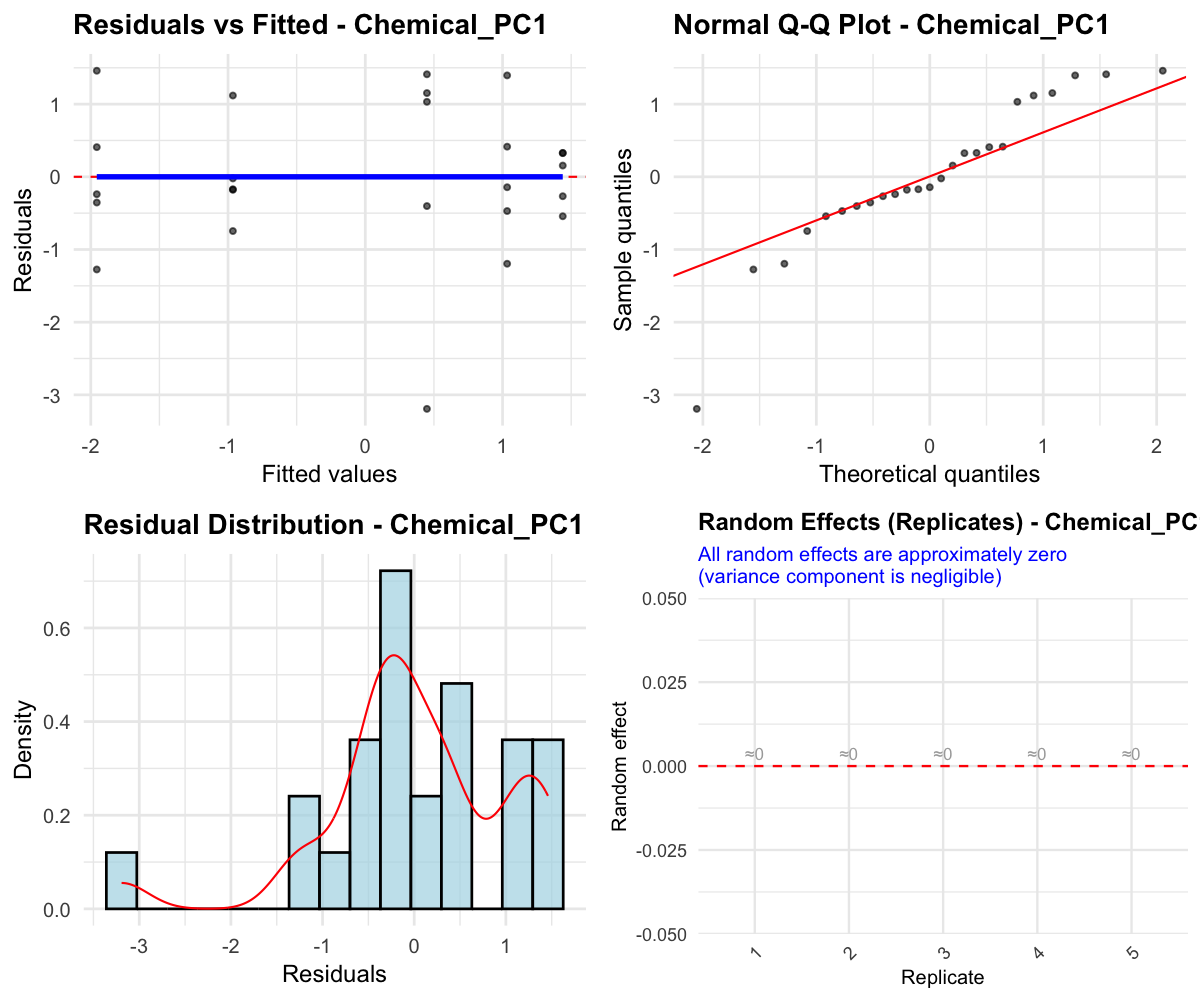


**Fig. S17.** Residual diagnostic plots for the mixed-effects model of the chemical composite indicator (PC1 score).


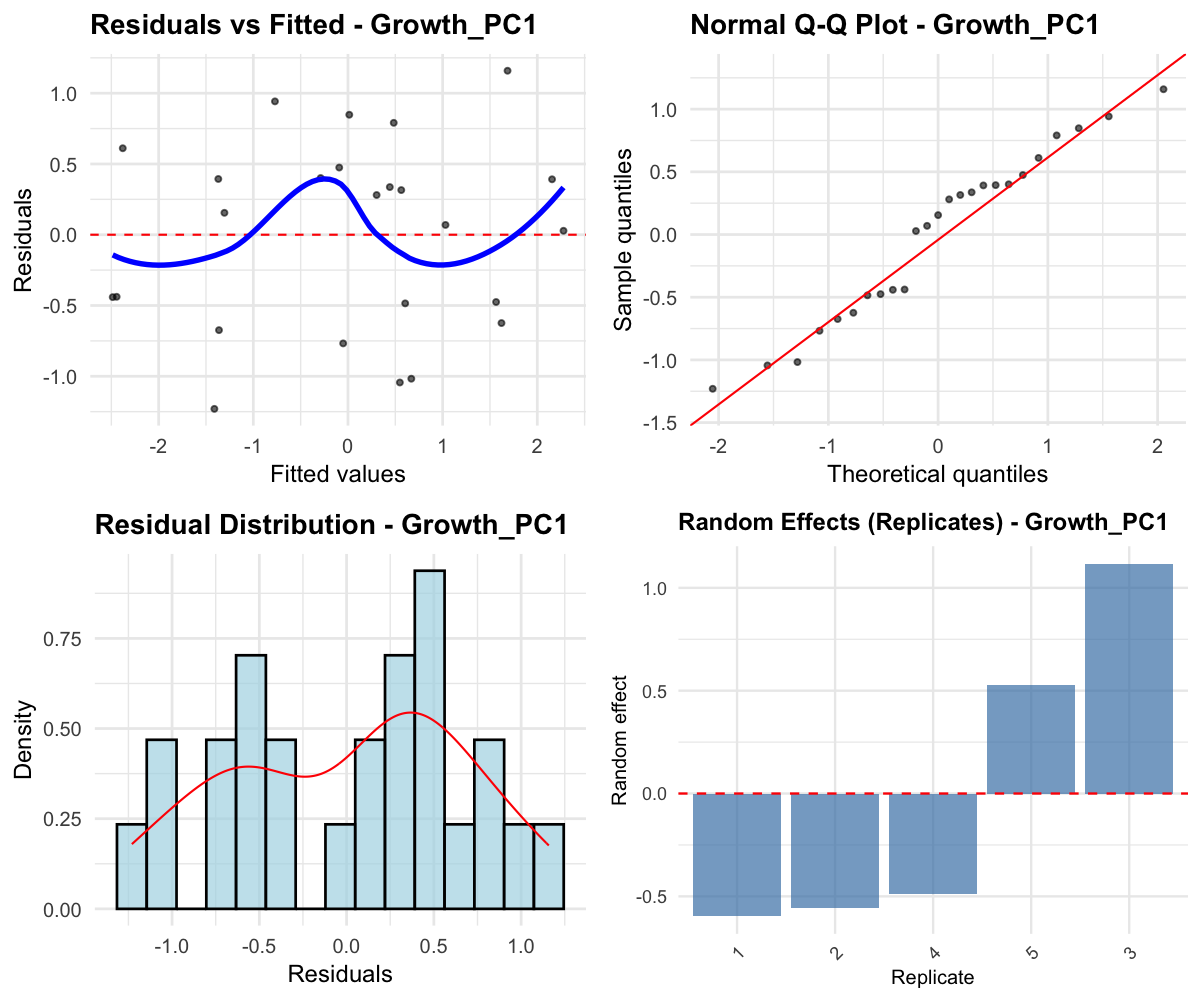


**Fig. S18.** Residual diagnostic plots for the mixed-effects model of the growth composite indicator (PC1 score).


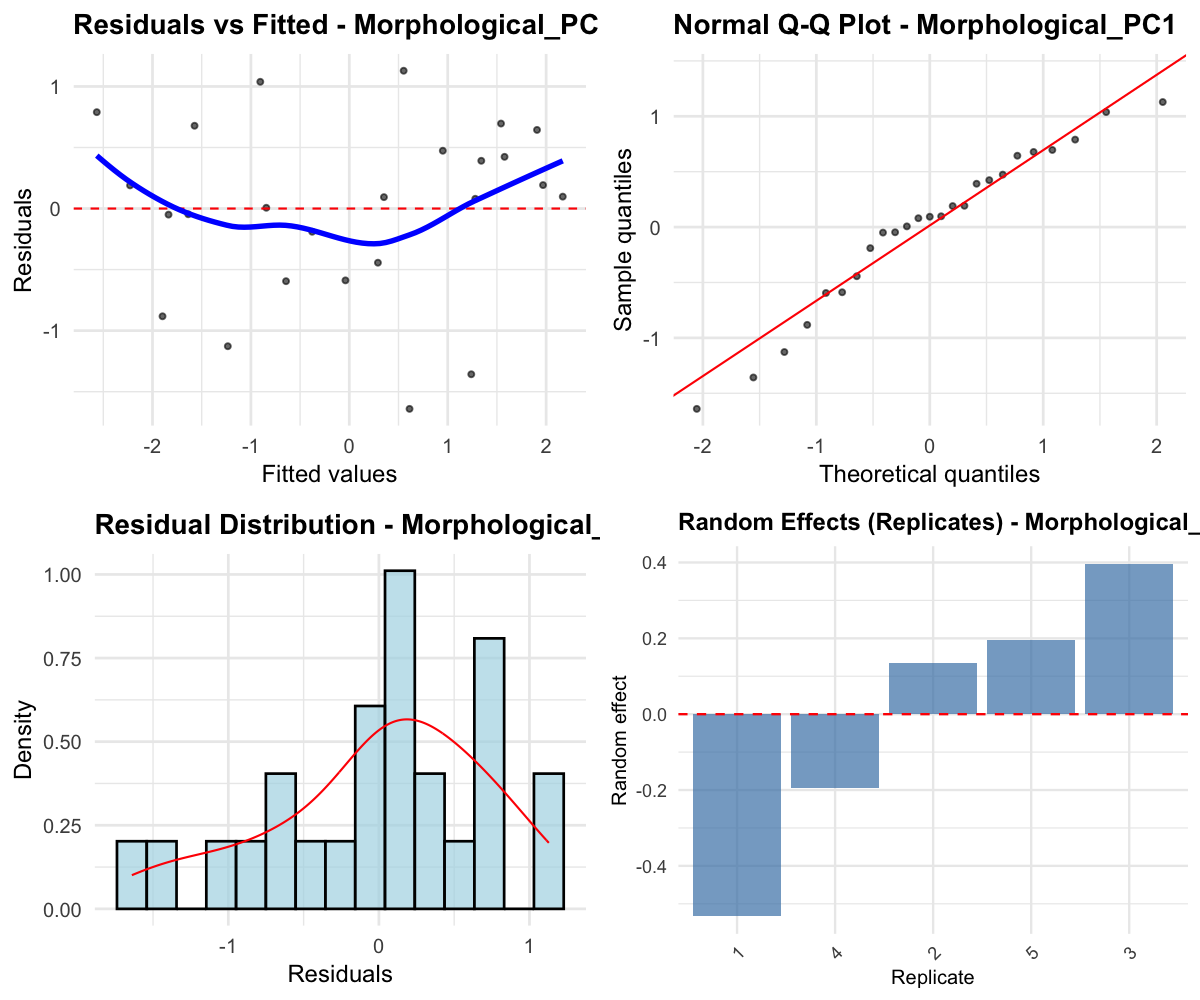


**Fig. S19.** Residual diagnostic plots for the mixed-effects model of the morphological composite indicator (PC1 score).

### 2.3 Part III: Correlation Analysis Among Indicators

#### 2.3.1 Normality Tests for Morphological Indicators

**Table S11.** Shapiro-Wilk normality test results for three morphological indicators (length, width, density) at three sprouting stages (early, middle, late).

| Variable | Stage | Shapiro_Wilk_W | Shapiro_Wilk_p | Normality_Interpretation |
| --- | --- | --- | --- | --- |
| Length | Early | 0.946 | 0.204 | Normal |
| Width | Early | 0.955 | 0.327 | Normal |
| Density | Early | 0.973 | 0.720 | Normal |
| Length | Middle | 0.988 | 0.985 | Normal |
| Width | Middle | 0.916 | 0.041 | Not Normal |
| Density | Middle | 0.971 | 0.673 | Normal |
| Length | Late | 0.985 | 0.963 | Normal |
| Width | Late | 0.927 | 0.073 | Normal |
| Density | Late | 0.937 | 0.127 | Normal |

**Table S12.** Shapiro-Wilk normality test results for all individual indicators used in comprehensive correlation analysis, including chemical quality indicators, final morphological indicators, and growth rate indicators.

| Variable | Sample_Size | Shapiro_Wilk_W | Shapiro_Wilk_p | Normal_Distribution |
| --- | --- | --- | --- | --- |
| Free amino acids | 25 | 0.9096 | 0.0298 | No |
| Tea polyphenols | 25 | 0.9828 | 0.934 | Yes |
| Phenol/aminoacid ratio | 25 | 0.9573 | 0.3625 | Yes |
| Bud length | 25 | 0.985 | 0.963 | Yes |
| Bud width | 25 | 0.9267 | 0.0731 | Yes |
| Bud number | 25 | 0.9371 | 0.1265 | Yes |
| Bud size | 25 | 0.961 | 0.4347 | Yes |
| Length growth | 25 | 0.9622 | 0.4603 | Yes |
| Width growth | 25 | 0.941 | 0.1564 | Yes |
| Number growth | 25 | 0.9204 | 0.0523 | Yes |
| Size growth | 25 | 0.9707 | 0.6637 | Yes |

#### 2.3.2 Bootstrap Tests for Correlation Differences

**Table S13.** Bootstrap test results comparing Spearman correlation coefficients between pairs of morphological indicators across different sprouting stages. Results include observed difference, 95% confidence intervals, p-values, and significance levels.

| Pair | Comparison | obs_diff | ci_lower | ci_upper | p_value | Significance | CI_95 |
| --- | --- | --- | --- | --- | --- | --- | --- |
| Length-Width | Early vs Mid | 0.258 | -0.214 | 0.733 | 0.270 | ns | [-0.214, 0.733] |
| Length-Width | Early vs Late | 0.765 | 0.215 | 1.217 | 0.008 | ** | [0.215, 1.217] |
| Length-Width | Mid vs Late | 0.507 | -0.026 | 1.004 | 0.062 | ns | [-0.026, 1.004] |
| Length-Density | Early vs Mid | 0.664 | 0.084 | 1.181 | 0.024 | * | [0.084, 1.181] |
| Length-Density | Early vs Late | 0.234 | -0.304 | 0.740 | 0.388 | ns | [-0.304, 0.740] |
| Length-Density | Mid vs Late | -0.430 | -0.968 | 0.176 | 0.164 | ns | [-0.968, 0.176] |
| Width-Density | Early vs Mid | 0.645 | 0.035 | 1.181 | 0.036 | * | [0.035, 1.181] |
| Width-Density | Early vs Late | 1.198 | 0.747 | 1.578 | 0.000 | *** | [0.747, 1.578] |
| Width-Density | Mid vs Late | 0.553 | 0.143 | 0.989 | 0.006 | ** | [0.143, 0.989] |
